# Efficient and flexible implementation of Langevin simulation for gene burst production

**DOI:** 10.1038/s41598-017-16835-y

**Published:** 2017-12-04

**Authors:** Ching-Cher Sanders Yan, Surendhar Reddy Chepyala, Chao-Ming Yen, Chao-Ping Hsu

**Affiliations:** 1 Institute of Chemistry, Academia Sinica, Taipei, 115 Taiwan; 2 Bioinformatics Program, Taiwan International Graduate Program, Institute of Information Science, Academia Sinica, Taipei, 115 Taiwan; 3 Institute of Biomedical Informatics, National Yang-Ming University, Taipei, 112 Taiwan; 4 Institute of Biochemical Sciences, College of Life Science, National Taiwan University, Taipei, 106 Taiwan; 5 Institute of Biological Chemistry, Academia Sinica, Taipei, 115 Taiwan; 6 Genome and Systems Biology Degree Program, National Taiwan University, Taipei, 106 Taiwan

## Abstract

Gene expression involves bursts of production of both mRNA and protein, and the fluctuations in their number are increased due to such bursts. The Langevin equation is an efficient and versatile means to simulate such number fluctuation. However, how to include these mRNA and protein bursts in the Langevin equation is not intuitively clear. In this work, we estimated the variance in burst production from a general gene expression model and introduced such variation in the Langevin equation. Our approach offers different Langevin expressions for either or both transcriptional and translational bursts considered and saves computer time by including many production events at once in a short burst time. The errors can be controlled to be rather precise (<2%) for the mean and <10% for the standard deviation of the steady-state distribution. Our scheme allows for high-quality stochastic simulations with the Langevin equation for gene expression, which is useful in analysis of biological networks.

## Introduction

Gene expression is a series of biochemical reactions that produce proteins for various biological functions. For cells with identical genes, gene expression noise is observed in both prokaryotes^1,2^ and eukaryotes^3,4^. One general source of such noise is from the probabilistic nature of chemical reactions, because the biological components involved in such reactions are in small copy numbers. In addition, as observed experimentally, both mRNAs^5^ and proteins^6^ are produced in discontinuous bursts of multiple copies in a short time, and thus, the corresponding fluctuation is increased^7^. Noise propagates through the biochemical networks^8^ and may further contribute to the heterogeneity in the phenotypes^9–11^. With the noise, fluctuation-dissipation theorem allows us to derive the dynamic response and infer dynamic properties in a cell^12^. When a precise control is needed, it may be necessary to reduce or buffer such noises^13–15^. Therefore, to gain insights into general biological processes by modeling, a good description for the fluctuation in gene expression is needed.

A complete accounting for the fluctuation in chemical reactions can be obtained by simulations with the Gillespie algorithm^16^. The Gillespie algorithm is a scheme that simulates every reaction event with a proper probability. Without imposing any additional approximations^17^, it generates trajectories that follow the exact probability distribution. Since each reaction involves only a small set of changes in molecular numbers, the process is time-consuming for a large system. To accelerate the simulation, a long leaping-time step can be used to account for several reaction events together. With slightly changed reaction propensities, a chemical Langevin equation can be derived^18^. Simulation is more efficient with the Langevin equation than the Gillespie algorithm. Moreover, the Langevin equation allows for a direct dissection and analysis of different noise sources^8,11^. It is therefore highly desirable to develop the Langevin equation for various biochemical processes.

**Figure 1:**
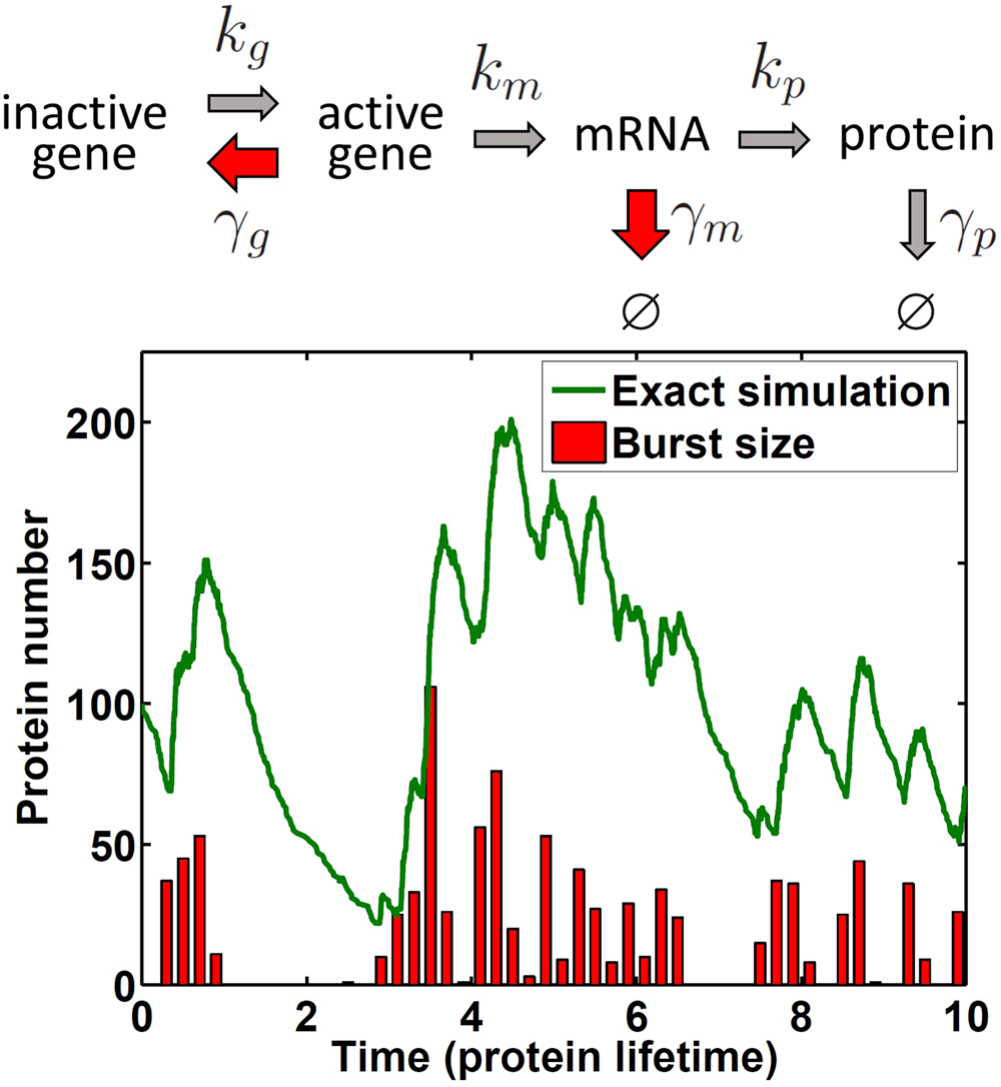
A general model of gene expression with burst productions and its stochastic dynamics of protein number. (a) The scheme of reactions for gene expression. (b) Shown are a stochastic trajectory (green) from the Gillespie algorithm, with the protein’s intermittent burst production indicated by red bars in time steps of 0.2 protein lifetime (1/*γ*_*p*_). Under the conditions applied, ${\gamma }_{g}\gg ({\gamma }_{m},{k}_{g})$ and ${\gamma }_{m}\gg {\gamma }_{p}$, rapid rises in the trajectory are seen, and protein production can be described as in bursts. Parameters used are *k*_*g*_ = 5, *γ*_*g*_ = 95, *k*_*m*_ = 200, *γ*_*m*_ = 10, *k*_*p*_ = 100 and *γ*_*p*_ = 1, which correspond to $\bar{p}=100$, average mRNA burst size ${\bar{b}}_{m}={k}_{m}/({k}_{g}+{\gamma }_{g})=2$ and protein average burst size ${\bar{b}}_{p}={k}_{p}/{\gamma }_{m}=10$.

To formulate a Langevin equation for gene expression, the burst properties need to be properly accounted for. Experiments found that for both mRNA and protein, the burst event can be described as a Poisson distribution, with the burst size as an exponential (or geometric) distribution. A general gene expression model^4,19,20^ shown in Fig. 1 allows us to define the burst frequency and the burst size in transcription and translation with fundamental rate constants^4,20–22^. Furthermore, the distributions of burst events and sizes derived from this model have the same features as those observed in experiments. The gene expression model shown in Fig. 1a can be written as:

1 $$\frac{dg}{dt}={k}_{g}(1-g)-{\gamma }_{g}g$$

2 $$\frac{dm}{dt}={k}_{m}g-{\gamma }_{m}m$$

3 $$\frac{dp}{dt}={k}_{p}m-{\gamma }_{p}p,$$

where *g* is the fraction of active gene for transcription and (*m*, *p*) are the amount of mRNA and protein, respectively; *k*_*g*_ and *γ*_*g*_ are the gene’s activation and deactivation rates; *k*_*m*_ and *k*_*p*_ are the production rates for mRNA and protein; and *γ*_*m*_ and *γ*_*p*_ are the corresponding degradation rates. Following previous works^21,22^, when ${\gamma }_{g}\gg ({\gamma }_{m},{k}_{g})$, mRNA production can be considered as occurring in bursts. Because the gene activation time (1/*γ*_*g*_) is rather short, the average amount of mRNAs produced in such short time interval is the mean burst size^22^:

4 $${\bar{b}}_{m}=\frac{{k}_{m}}{{\gamma }_{g}}\mathrm{.}$$

The low gene activation rate (*k*_*g*_) leads to well-separated burst events. The *k*_*g*_ is considered the mRNA burst frequency. Similar limiting set $({\gamma }_{m}\gg {\gamma }_{p})$ ^7,20^ applies to protein production, leading to an average burst size of protein as

5 $${\bar{b}}_{p}=\frac{{k}_{p}}{{\gamma }_{m}},$$

and burst frequency as the rate of mRNA production (*g*(*t*)*k*_*m*_). In Fig. 1b, we include a stochastic trajectory under the limit of burst-like production. In this work, we aimed to derive a Langevin equation that includes burst production effects and offers good number fluctuation for gene expression.

In the burst regime, when the upstream component is rarely-produced and fast-degraded, the slowly-degraded downstream component would be produced in bursts. Such difference in rates poses a difficulty for simulations with both the Gillespie algorithm and the standard Langevin equation. For the Gillespie algorithm, the slow reactions are sampled rarely, which leads to poor statistics. The Langevin simulation efficiency is also reduced, because the time step size has to be adjusted for the fast changes of the gene switching or mRNA number fluctuation. Therefore, we need a Langevin equation for the protein fluctuation that does not have to track the fast changes of a gene’s state or mRNA’s number^23,24^.

Starting from the general model, we develop analytical expressions for the mean and variance in the production with the burst effect, and such expression is included in the Langevin equation. Our approach allows for the flexibility to include either or both of the mRNA’s and protein’s burst effects. We also found that our burst Langevin expression has a large applicable region, which is not limited by the case of burst production. Our algorithm can produce an accurate steady-state mean and similar distribution as that with Gillespie simulation. When a gene switches dynamically, our simulation also can produce accurate dynamics of average protein number. The burst Langevin equation we derived is effective in minimizing the computational time and memory in stochastic simulations. Our simulation scheme with the burst Langevin equation is useful in stochastic simulation for biological networks.

## Theory

### Langevin equation for burst production

To simplify the derivation of burst Langevin equation, we first consider a two-component model for the burst of either mRNA or protein. In this model, a short-lived *x* results in a burst event of *y*:

6 $$\frac{dx}{dt}={k}_{x}-{\gamma }_{x}x$$

7 $$\frac{dy}{dt}={k}_{y}x-{\gamma }_{y}y.$$

For the mRNA’s burst production in equations (1) and (2), we assign *x* as the state of the gene and *y* as the mRNA. In this case, we can combine the terms *k*_*g*_ + *γ*_*g*_ and set it to *γ*_*x*_. Similarly, for the protein’s burst production, *x* is mRNA and *y* is protein. We treat *g* as a constant in equation (2) for a constant mRNA production rate and set *k*_*m*_ *g* as *k*_*x*_. Thus, both the mRNA’s and protein’s production can be described by equations (6) and (7).

To develop an efficient stochastic simulation, we select a time interval *τ* that is longer than *x*’s lifetime (1/*γ*_*x*_). When there are *e*_*y*_ burst events and each burst size is denoted as *b*_*yl*_, the change in *y* is:

8 $$y(t+\tau )=y(t)+\sum _{l=1}^{{e}_{y}}{b}_{yl}-[{\gamma }_{y}y\tau +{({\gamma }_{y}y\tau )}^{1/2}{{\mathscr{N}}}_{2}(0,1)].$$

The burst production of *y* is the consequence of short-lived *x*. The number of burst events (*e*_*y*_) is determined by the number of *x* produced in *τ* and each burst size (*b*_*yl*_) is determined by the survival time of each *x*. Simulation for the production in equation (8) can be performed with a random number for *e*_*y*_, followed by several random numbers for various burst sizes *b*_*yl*_. For the degradation in *τ*, a Poisson distribution can be used, with both mean and variance being *γ*_*y*_ *yτ* ^18^. A Gaussian random number with zero mean and unit variance ${{\mathscr{N}}}_{2}\mathrm{(0,}\,\mathrm{1)}$ is scaled by the standard deviation (*γ*_*y*_ *yτ*)^1/2^ for the noise part of degradation. An alternative approach is to reformulate the production of *y* in *τ* as:

9 $$y(t+\tau )=y(t)+[{{\rm{\Delta }}}_{y}(\tau )+{\sigma }_{{\Delta }_{y}}(\tau ){{\mathscr{N}}}_{1}(0,1)]-[{\gamma }_{y}y\tau +{({\gamma }_{y}y\tau )}^{1/2}{{\mathscr{N}}}_{2}(0,1)],$$

where Δ_*y*_(*τ*) and σ_Δ*y*_(*τ*) are the mean and standard deviation of *y*’s production within time *τ*. In this way, the simulation steps are simplified, and the computation is more efficient. To estimate Δ_*y*_(*τ*), the average production of *y* in time *τ*, we assumed that burst events and burst sizes are independent random processes. Therefore, we can take their average separately:

10 $$\begin{array}{rcl}{\Delta }_{y}(\tau ) & = & \langle \sum _{l\mathrm{=1}}^{{e}_{y}}{b}_{yl}\rangle =\langle \sum _{l\mathrm{=1}}^{{e}_{y}}\langle {b}_{yl}\rangle \rangle =\langle \sum _{l\mathrm{=1}}^{{e}_{y}}{\bar{b}}_{y}\rangle \\  & = & \langle {e}_{y}\rangle {\bar{b}}_{y}={\bar{e}}_{y}{\bar{b}}_{y},\end{array}$$

which is the product of average burst event $({\bar{e}}_{y})$ and average burst size $({\bar{b}}_{y})$.

The variance of *y*’s production distribution in time *τ*, ${\sigma }_{{{\rm{\Delta }}}_{y}}^{2}(\tau )$, was derived from the characteristic function of *P*(*y*), the probability distribution of *y*’s number, in the supplementary material of ref.^25^:

11 $${\sigma }_{{{\rm{\Delta }}}_{y}}^{2}(\tau )={\bar{e}}_{y}{\sigma }_{by}^{2}+{\sigma }_{ey}^{2}{\bar{b}}_{y}^{2},$$

where ${\sigma }_{by}^{2}$ is the variance of burst size and ${\sigma }_{ey}^{2}$ is that of burst event number in time *τ*. We found that it can also be derived directly,

12 $$\begin{array}{rcl}{\sigma }_{{{\rm{\Delta }}}_{y}}^{2}(\tau ) & = & \langle {{\rm{\Delta }}}_{y}^{2}\rangle -{\langle {{\rm{\Delta }}}_{y}\rangle }^{2}\\  & = & \langle {(\sum _{l\mathrm{=1}}^{{e}_{y}}{b}_{yl})}^{2}\rangle -{\langle \sum _{l\mathrm{=1}}^{{e}_{y}}{b}_{yl}\rangle }^{2}\\  & = & \langle \sum _{l\mathrm{=1}}^{{e}_{y}}{b}_{yl}^{2}+2\sum _{1\le l < l^{\prime} }^{{e}_{y}}{b}_{yl}{b}_{yl^{\prime} }\rangle -{\bar{e}}_{y}^{2}{\bar{b}}_{y}^{2}\\  & = & {\bar{e}}_{y}\langle {b}_{yl}^{2}\rangle +2\frac{\langle {\bar{e}}_{y}({\bar{e}}_{y}-1)\rangle }{2}\langle {b}_{yl}{b}_{yl^{\prime} }\rangle -{\bar{e}}_{y}^{2}{\bar{b}}_{y}^{2}\mathrm{.}\end{array}$$

With the same assumption that different processes are independent, ${\bar{e}}_{y}$ and $\langle {\bar{e}}_{y}({\bar{e}}_{y}-1)\rangle$ can be separated from $\langle {b}_{yl}^{2}\rangle$ and $\langle {b}_{yl}{b}_{yl^{\prime} }\rangle$, respectively. We also replaced $\langle {b}_{yl}{b}_{yl^{\prime} }\rangle$ with ${\bar{b}}_{y}^{2}$ by assuming different bursts are independent. With the definition of variance, we also replaced $\langle {b}_{yl}^{2}\rangle$ with ${\sigma }_{by}^{2}+{\bar{b}}_{y}^{2}$ and $\langle {\bar{e}}_{y}^{2}\rangle -{\bar{e}}_{y}^{2}$ with ${\sigma }_{ey}^{2}$. Therefore, we obtain the same variance expression for *y*’s burst production as in ref.^25^ by direct estimation.

To simulate the downstream *y*’s fluctuation with burst production, we can follow the Langevin equation as in equation (9) including the mean propagation Δ_*y*_(*τ*) as given in equation (10) and variance ${\sigma }_{{{\rm{\Delta }}}_{y}}^{2}(\tau )$ as in equation (11). The expressions derived in this section can be applied to either or both the mRNA’s and protein’s burst production.

### Langevin equations for either or both mRNA and protein bursts

**Figure 2:**
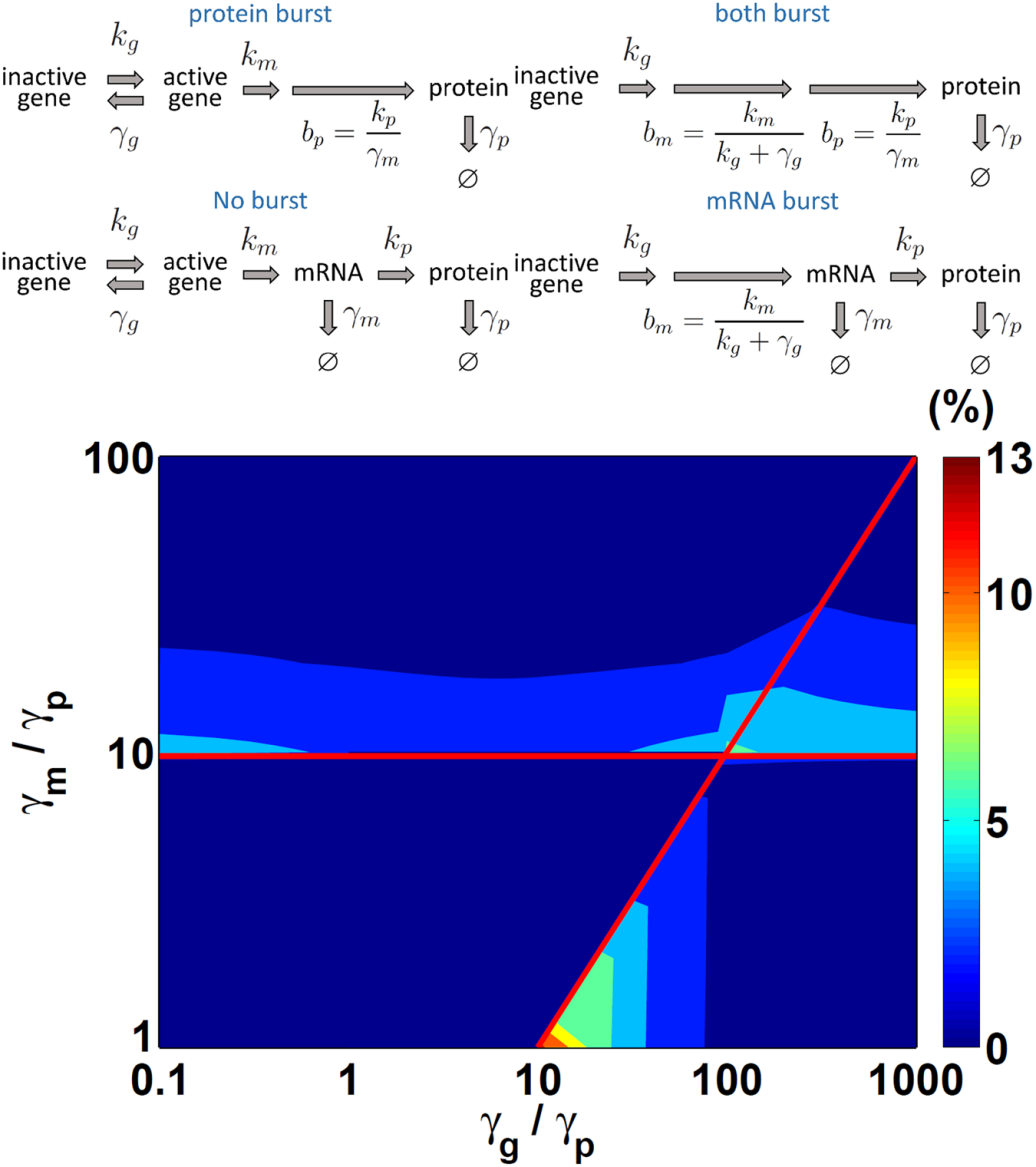
Four cases of gene expression dynamics and errors from different Langevin equations. (a) Four possible cases of gene expression. When an activated gene state is short-lived, the Langevin equation skips the tracking for the gene state, and a burst production following the statistics is used for mRNA. Similarly, when the mRNA’s lifetime is short, burst production of protein is introduced, instead of tracking the mRNA. (b) Shown are normalized errors (%) of a steady-state protein’s standard deviation (${\sigma }_{p,ss}$) from the burst Langevin equations compared to the squared root of exact variance expression as in equation (26), as a function of gene deactivation rate *γ*_*g*_ and mRNA degradation rates *γ*_*m*_. Red lines are the boundaries for the four different cases in the burst models. Other parameters are $\bar{p}=100$, *k*_*g*_ = 5, mRNA burst size ${\bar{b}}_{m}=2$, protein burst size ${\bar{b}}_{p}=10$, and *γ*_*p*_ = 1.

Generally, different genes may have different dynamic behaviors depending on their degradation rates. Some genes in mammalian cells have only obvious mRNA burst production $({\gamma }_{g}\gg {\gamma }_{m}\sim {\gamma }_{p})$ ^26,27^, whereas some genes in yeast have only protein burst production $({\gamma }_{m}\gg {\gamma }_{g}\sim {\gamma }_{p})$ ^4^. Furthermore, some genes in bacteria have both mRNA and protein bursts $({\gamma }_{g}\gg {\gamma }_{m}\gg {\gamma }_{p})$ and some do not have any obvious burst production $({\gamma }_{g}\sim {\gamma }_{m}\sim {\gamma }_{p})$ ^28^. We further explored the criteria of *γ*_*g*_ and *γ*_*m*_ comparing to *γ*_*p*_ with and without burst production, as shown in Fig. 2a. For all these different burst cases, we show that the burst production variance in equation (11) has the flexibility to describe all of them.

#### mRNA burst

With the condition $({\gamma }_{g}\gg {\gamma }_{m}\sim {\gamma }_{p})$, only mRNA is generated in bursts. We rearranged the expression in equation (1) as:

13 $$\frac{dg}{dt}={k}_{g}-({k}_{g}+{\gamma }_{g})g,$$

which becomes identical to equation (6). For a single-copy gene, the activity fraction *g* in equation (13) is considered 0 or 1 for off or on state, respectively. Without loss of generality, we considered a single-copy gene in the present work. If the gene has *n*-copies, $(1-g)$ in equation (1) can be replaced by $(n-g)$; thus, the first *k*_*g*_ in equation (13) needs to be replaced by *nk*_*g*_.

mRNA burst frequency is equal to *k*_*g*_ (or *nk*_*g*_ for *n*-copy gene case). Assuming that each mRNA burst event is independent, we can approximate burst event distribution by a Poisson distribution, as observed in several experiments^5,22^, where the mean of burst events $({\bar{e}}_{m}={k}_{g}\tau )$ equals the variance $({\sigma }_{em}^{2})$:

14 $${\bar{e}}_{m}={k}_{g}\tau ={\sigma }_{em}^{2}.$$

On the other hand, possible mRNA burst size (*b*_*m*_) can be described by a geometric distribution^29^,

15 $$P({b}_{m})=q{(1-q)}^{{b}_{m}},$$

where *q* is the probability of no mRNA produced from this activation period, thus, *q* is proportional to *k*_*g*_ + *γ*_*g*_. One mRNA is produced with the probability of (1 − *q*), which is proportional to *k*_*m*_, the transcription rate constant in equation (2). The mean and variance of mRNA burst size are

16 $${\bar{b}}_{m}=\frac{1-q}{q}=\frac{{k}_{m}}{{k}_{g}+{\gamma }_{g}}$$

17 $${\sigma }_{bm}^{2}={\bar{b}}_{m}^{2}+{\bar{b}}_{m}.$$

We note that the mRNA burst size definition is modified as in equation (16), instead of ${\bar{b}}_{m}={k}_{m}/{\gamma }_{g}$ in the literature which is obtained with very small *k*_*g*_^21,22^. This new definition for ${\bar{b}}_{m}$ yields accurate kinetic expression for the average amount of mRNA, as production (burst frequency (*k*_*g*_) multiplied by size $({\bar{b}}_{m})$) divided by degradation rate constant (*γ*_*m*_):

18 $$\bar{m}=\frac{{k}_{g}{\bar{b}}_{m}}{{\gamma }_{m}}.$$

These are the statistical features of burst distributions that needs to be included in the Langevin equation of mRNA burst.

The mean of mRNA production with bursts (with large *γ*_*g*_) can be expressed as:

19 $${{\rm{\Delta }}}_{m}(\tau )={\bar{e}}_{m}{\bar{b}}_{m}={k}_{g}\tau {\bar{b}}_{m}.$$

by following equation (10). The variance for mRNA is

20 $$\begin{array}{rcl}{\sigma }_{{{\rm{\Delta }}}_{m}}^{2}(\tau ) & = & {\bar{e}}_{m}{\sigma }_{bm}^{2}+{\sigma }_{em}^{2}{\bar{b}}_{m}^{2}\\  & = & {k}_{g}\tau ({\bar{b}}_{m}^{2}+{\bar{b}}_{m})+{k}_{g}\tau {\bar{b}}_{m}^{2}\\  & = & {k}_{g}\tau {\bar{b}}_{m}(2{\bar{b}}_{m}+1),\end{array}$$

by following equation (11). With equations (19) and (20), the Langevin equation for mRNA is then

21 $$\begin{array}{rcl}m(t+\tau ) & = & m(t)+({k}_{g}\tau {\bar{b}}_{m}+{({k}_{g}\tau {\bar{b}}_{m}(2{\bar{b}}_{m}+1))}^{\mathrm{1/2}}{{\mathscr{N}}}_{1}\mathrm{(0,}\,\mathrm{1)})\\  &  & -\,({\gamma }_{m}m\tau +{({\gamma }_{m}m\tau )}^{\mathrm{1/2}}{{\mathscr{N}}}_{2}\mathrm{(0,}\,\mathrm{1)}),\end{array}$$

and following the amount of mRNA, the Langevin equation for protein is

22 $$\begin{array}{rcl}p(t+\tau ) & = & p(t)+({k}_{p}m\tau +{({k}_{p}m\tau )}^{\mathrm{1/2}}{{\mathscr{N}}}_{3}\mathrm{(0,}\,\mathrm{1)})\\  &  & -\,({\gamma }_{p}p\tau +{({\gamma }_{p}p\tau )}^{\mathrm{1/2}}{{\mathscr{N}}}_{4}\mathrm{(0,}\,\mathrm{1)}),\end{array}$$

where the gene state is skipped. From the mRNA’s burst Langevin equation in equation (21), we derived the mRNA’s steady-state variance as:

23 $${\sigma }_{m,ss}^{2}\approx \bar{m}({\bar{b}}_{m}+1).$$

by following the supplementary material of ref.^8^. The steps are transforming *m*(*t*) in equation (21) to the Fourier space first and then squaring, averaging, and finally inverse Fourier transforming. The detailed derivation is in the supplementary information of this work. By comparing the production variance in equation (20) and steady-state variance in equation (23), we can see that the *τ* in equation (20) is replaced by 1/*γ*_*m*_, leading to ${k}_{g}{\bar{b}}_{m}/{\gamma }_{m}=\bar{m}$ in equation (23). Also, the $2{\bar{b}}_{m}$ in equation (20) becomes ${\bar{b}}_{m}$ in equation (23). However, the mRNA’s exact variance expression in the steady state from linear noise approximation (LNA)^30–32^ is

24 $${\sigma }_{m,ss}^{2}=\bar{m}(\frac{{\gamma }_{g}}{{k}_{g}+{\gamma }_{g}+{\gamma }_{m}}\frac{{k}_{m}}{{k}_{g}+{\gamma }_{g}}+1)=\bar{m}(\frac{{\gamma }_{g}}{{k}_{g}+{\gamma }_{g}+{\gamma }_{m}}{\bar{b}}_{m}+1),$$

with detailed derivation given in our supplementary information. By comparing equations (23) and (24), we can see that the burst Langevin approximation can be achieved by assuming *γ*_*g*_/(*k*_*g*_ + *γ*_*g*_ + *γ*_*m*_) ≈ 1 in the LNA’s result, which is true that a large *γ*_*g*_ leads to mRNA bursts. Therefore, with equations (21) and (22), there is no need to track the fast-changing gene state *g*(*t*) in the simulation, and a modest error is introduced in the mRNA’s variance as in equation (23).

To further calculate the protein’s steady-state variance, because of ${\gamma }_{m}\sim {\gamma }_{p}$, we can propagate ${\sigma }_{m,ss}^{2}$ from equation (23) by the variance propagation equation^33^ to obtain ${\sigma }_{p,ss}^{2}$:

25 $$\begin{array}{rcl}{\sigma }_{p,ss}^{2} & = & {\sigma }_{m,ss}^{2}\frac{{k}_{p}}{{\gamma }_{p}}\frac{{k}_{p}}{{\gamma }_{m}+{\gamma }_{p}}+\bar{p}\\  & = & \bar{p}({\bar{b}}_{m}\frac{{k}_{p}}{{\gamma }_{m}+{\gamma }_{p}}+\frac{{k}_{p}}{{\gamma }_{m}+{\gamma }_{p}}+1)\mathrm{.}\end{array}$$

This expression is also slightly different from the exact expression derived from LNA (details in the supporting information), which is given below:

26 $${\sigma }_{p,ss}^{2}=\bar{p}(\frac{{\gamma }_{g}({\gamma }_{g}+{\gamma }_{m}+{\gamma }_{p}+{k}_{g})}{({\gamma }_{g}+{\gamma }_{m}+{k}_{g})({\gamma }_{g}+{\gamma }_{p}+{k}_{g})}\frac{{k}_{m}}{{k}_{g}+{\gamma }_{g}}\frac{{k}_{p}}{{\gamma }_{m}+{\gamma }_{p}}+\frac{{k}_{p}}{{\gamma }_{m}+{\gamma }_{p}}+1).$$

In general, ${\sigma }_{p,ss}^{2}$ obtained by LNA as in equation (26) includes the overall intrinsic noise of a gene following equations (1) to (3). So it is desirable to compare the *σ* ^2^_*p*,*ss*_ in equation (25) from the burst Langevin equation to the exact variance in equation (26). The difference between equations (25) and (26) gives us an indication of the burst Langevin equation’s accuracy. Such difference is shown in the lower right region in Fig. 2b, where *γ*_*g*_ ≥ 10 *γm*. The largest error of the bursting Langevin equation with mRNA burst alone is $\mathrm{12.5 \% }$ that occurs at the lower left boundary of the region, which is still acceptable.

#### Both mRNA and protein bursts

In the condition ${\gamma }_{g}\gg {\gamma }_{m}\gg {\gamma }_{p}$, both the mRNA’s and protein’s production are produced in bursts. We can combine both bursts and derive one Langevin equation for the protein’s fluctuation, thereby greatly simplifying the simulation. Because each mRNA corresponds to a protein burst event, the number of protein burst events in *τ* is

27 $${\bar{e}}_{p}={k}_{g}{\bar{b}}_{m}\tau ,$$

leading to the protein production as

28 $${{\rm{\Delta }}}_{p}={k}_{g}{\bar{b}}_{m}{\bar{b}}_{p}\tau .$$

Since mRNA is also produced in bursts, the variance of the protein’s burst event is

29 $${\sigma }_{ep}^{2}={\sigma }_{{{\rm{\Delta }}}_{m}}^{2}={k}_{g}{\bar{b}}_{m}\tau (2{\bar{b}}_{m}+1),$$

which is identical to that in equation (20). The variance of the protein’s production following equation (11) is

30 $$\begin{array}{rcl}{\sigma }_{{{\rm{\Delta }}}_{p}}^{2} & = & {\bar{e}}_{p}{\sigma }_{bp}^{2}+{\sigma }_{ep}^{2}{\bar{b}}_{p}^{2}\\  & = & {k}_{g}{\bar{b}}_{m}\tau ({\bar{b}}_{p}^{2}+{\bar{b}}_{p})+{k}_{g}{\bar{b}}_{m}\tau (2{\bar{b}}_{m}+1){\bar{b}}_{p}^{2}\\  & = & {k}_{g}{\bar{b}}_{m}\tau {\bar{b}}_{p}(2{\bar{b}}_{m}{\bar{b}}_{p}+2{\bar{b}}_{p}+1).\end{array}$$

We note that ${\sigma }_{bp}^{2}$ is equal to ${\bar{b}}_{p}^{2}+{\bar{b}}_{p}$, by following the same assumption of geometric distribution as ${\bar{b}}_{m}$ in equation (15). We also note that similar results with both bursts were derived using the generation function of *P*(*p*), the probability distribution of *p*’s number in the supplementary material of ref.^34^.

With equations (28) and (30), the Langevin equation for protein fluctuation with both bursts is

31 $$\begin{array}{rcl}p(t+\tau ) & = & p(t)+({k}_{g}{\bar{b}}_{m}{\bar{b}}_{p}\tau +{({k}_{g}{\bar{b}}_{m}{\bar{b}}_{p}\tau (2{\bar{b}}_{m}{\bar{b}}_{p}+2{\bar{b}}_{p}+1))}^{1/2}{{\mathscr{N}}}_{1}(0,1))\\  &  & -({\gamma }_{p}p\tau +{({\gamma }_{p}p\tau )}^{1/2}{{\mathscr{N}}}_{2}(0,1)).\end{array}$$

It allows us to efficiently simulate protein’s fluctuation, because we can skip tracking the gene state and the mRNA in the simulation.

Following the same process as we obtained the mRNA’s steady-state variance ${\sigma }_{m,ss}^{2}$ as in equation (23), here we obtained the protein’s steady-state variance from equation (31) as

32 $${\sigma }_{p,ss}^{2}\approx \bar{p}({\bar{b}}_{m}{\bar{b}}_{p}+{\bar{b}}_{p}+1).$$

In the upper right region of Fig. 2b, we show the normalized error in ${\sigma }_{p,ss}$ for equation (32) to that from LNA as equation (26) with the condition of both bursts as following:

33 $${\gamma }_{g}\ge 10\,{\gamma }_{m}\ge 100\,{\gamma }_{p}.$$

The largest possible error in the standard deviation is <6.5%, which is quite acceptable.

#### Protein burst

When the gene’s active state is long-lived $({\gamma }_{g}\sim {\gamma }_{p})$, the mRNA is not produced in bursts. For some genes $({\gamma }_{m}\gg {\gamma }_{g}\sim {\gamma }_{p})$ reported in yeast^4^, short-lived mRNA leads to the protein’s burst production. Partial simplification of the Langevin equations for the gene expression is still possible if the protein is produced in bursts. For such case, we keep track the gene’s activity, skip the short-lived mRNA, and develop the protein’s Langevin equation with bursts:

34 $$\begin{array}{ccc}g(t+\tau ) & = & g(t)+{k}_{g}(1-g)\tau -{\gamma }_{g}g\tau \end{array}$$

35 $$p(t+\tau )=p(t)+(g{k}_{m}\tau {\bar{b}}_{p}+{\sigma }_{{\rm{\Delta }}p}{{\mathscr{N}}}_{1}(0,1))-({\gamma }_{p}p\tau +({\gamma }_{p}p\tau {)}^{1/2}{{\mathscr{N}}}_{2}(0,1))$$

The gene-switching probability is *k*_*g*_ *τ* with *g* = 0 and *γ*_*g*_ *τ* with *g* = 1. The mean production of the protein number in time *τ* is $g{k}_{m}\tau {\bar{b}}_{p}$, which is *gk*_*m*_ *τ*, the number of protein burst events (same as the number of mRNA molecules) produced in *τ*, multiplied by ${\bar{b}}_{p}$, the mean protein burst size. For the noise strength (*σ*_Δ*p*_) in equation (35), *g*(*t*)*k*_*m*_ can be considered a constant in *τ* because the state of the gene does not switch frequently in *τ*. Therefore, the mRNA produced or the protein burst event from equation (35) is a Poisson distribution, with the variance being the same as the mean:

36 $${\sigma }_{ep}^{2}={\sigma }_{{\rm{\Delta }}m}^{2}=g(t){k}_{m}\tau .$$

Following equation (11), the variance of protein production in equation (35)can be written as

37 $$\begin{array}{rcl}{\sigma }_{{{\rm{\Delta }}}_{p}}^{2} & = & {\bar{e}}_{p}{\sigma }_{bp}^{2}+{\sigma }_{ep}^{2}{\bar{b}}_{p}^{2}\\  & = & g(t){k}_{m}\tau ({\bar{b}}_{p}^{2}+{\bar{b}}_{p})+g(t){k}_{m}\tau {\bar{b}}_{p}^{2}\\  & = & g(t){k}_{m}\tau {\bar{b}}_{p}(2{\bar{b}}_{p}+1).\end{array}$$

In a simulation trial, the noise strength of protein’s production $({\sigma }_{{\Delta }_{p}})$ follows the state of *g*(*t*). Also, because *g*(*t*) = 1 in some *τ* steps and *g*(*t*) = 0 in others, the average gene state is $\bar{g}={k}_{g}/({k}_{g}+{\gamma }_{g})$.

With only protein bursts, based on the condition of ${\gamma }_{m}\ge 10{\gamma }_{p}$, we can simplify ${\sigma }_{p,ss}^{2}$ in equation (26) from LNA to

38 $${\sigma }_{p,ss}^{2}\approx \bar{p}(\frac{{\gamma }_{g}}{{k}_{g}+{\gamma }_{g}+{\gamma }_{p}}{\bar{b}}_{m}{\bar{b}}_{p}+{\bar{b}}_{p}+1),$$

by reducing the first fraction and using ${\bar{b}}_{p}={k}_{p}/{\gamma }_{m}$. In the upper left region of Fig. 2b, comparison of *σ*_*p*,*ss*_ from equation (38) to that from the exact variance in equation (26) shows that the largest possible error is <5%.

In a special condition, where *k*_*g*_ and *γ*_*g*_ are significantly smaller than other four kinetic parameters in the gene expression model (equations (1) to (3)), bimodal distribution of the protein number can be obtained from the numerical simulation. Such parameter sets lie on the upper and most-left region of Fig. 2b, where the error of *σ*_*p*,*ss*_ from the burst Langevin is small as <5%. Considering *k*_*g*_ = *γ*_*g*_ = 0.1*γ*_*p*_ with *γ*_*m*_ = 10*γ*_*p*_, which leads to only protein bursts, the burst Langevin algorithm can fairly reproduce the bimodal distributions of the protein number with various combinations of *k*_*m*_ and ${\bar{b}}_{p}$. The comparison of distributions between the protein burst Langevin simulation and Gillespie algorithm in this special condition are shown in the supplementary information.

Overall, our comparison shows that the burst Langevin equation can provide reliable estimations of *σ*_*p*,ss_ for all three cases, where bursts are observed in mRNA, protein or both mRNA and protein. For the three cases, we organized the statistical expressions including burst events, burst sizes, variance of production and steady-state variance in Table 1. For three cases of bursts, the variance of the burst event as in equation (11) needs to be modified accordingly.

**Table 1:** Summary of statistics for three different cases of burst in gene expression.

	mRNA burst (*y* = *m*)	both bursts (*y* = *p*)	protein burst (*y* = *p*)
condition	*γ*_*g*_ ≥ 10 *γ*_*m*_	*γ*_*g*_ ≥ 10 *γ*_*m*_ ≥ 100 *γ*_*p*_	*γ*_*m*_ ≥ 10 *γ*_*p*_
simulated subjects	*m*(*t*), *p*(*t*)	*p*(*t*)	*g*(*t*), *p*(*t*)
burst event distribution:			
${\bar{e}}_{y}(\tau )$	*k*_*g*_ *τ*	${k}_{g}\tau {\bar{b}}_{m}$ ^‡^	*g*(*t*)*k*_*m*_ *τ*
${\sigma }_{ey}^{2}(\tau )$	*k*_*g*_ *τ*	${k}_{g}\tau {\bar{b}}_{m}(2{\bar{b}}_{m}+1)$ ^‡^	*g*(*t*)*k*_*m*_ *τ*
burst size^†^ distribution:			
${\bar{b}}_{y}$	${\bar{b}}_{m}$	${\bar{b}}_{p}$	${\bar{b}}_{p}$
${\sigma }_{by}^{2}$	${\bar{b}}_{m}^{2}+{\bar{b}}_{m}$	${\bar{b}}_{p}^{2}+{\bar{b}}_{p}$	${\bar{b}}_{p}^{2}+{\bar{b}}_{p}$
burst production in Langevin equation:			
${{\rm{\Delta }}}_{y}(\tau )$ by equation (10)	${k}_{g}\tau {\bar{b}}_{m}$‡	${k}_{g}\tau {\bar{b}}_{m}{\bar{b}}_{p}$	$g(t){k}_{m}\tau {\bar{b}}_{p}$
${\sigma }_{{{\rm{\Delta }}}_{y}}^{2}(\tau )$ by equation (11)	${k}_{g}\tau {\bar{b}}_{m}(2{\bar{b}}_{m}+1)$‡	${k}_{g}\tau {\bar{b}}_{m}{\bar{b}}_{p}(2{\bar{b}}_{m}{\bar{b}}_{p}+2{\bar{b}}_{p}+1)$	$g(t){k}_{m}\tau {\bar{b}}_{p}(2{\bar{b}}_{p}+1)$
steady-state distribution: exact expression^*^			
$\bar{m}\,=$ $\bar{g}\frac{{k}_{m}}{{\gamma }_{m}}$	same as exact	—	—
${\sigma }_{m,ss}^{2}\,=$ $\bar{m}({F}_{1}{\bar{b}}_{m}+1)$	$\bar{m}({\bar{b}}_{m}+1)$ ^§^	—	—
$\bar{p}\,=$ $\bar{m}\frac{{k}_{p}}{{\gamma }_{p}}$	same as exact	same as exact	same as exact
${\sigma }_{p,ss}^{2}\,=$ $\bar{p}({F}_{0}{\bar{b}}_{m}{\bar{b}}_{p0}+{\bar{b}}_{p0}+1)$	$\bar{p}({\bar{b}}_{m}{\bar{b}}_{p0}+{\bar{b}}_{p0}+1)$ ^¶^	$\bar{p}({\bar{b}}_{m}{\bar{b}}_{p}+{\bar{b}}_{p}+1)$ ^||^	$\bar{p}({F}_{2}{\bar{b}}_{m}{\bar{b}}_{p}+{\bar{b}}_{p}+1)$ ^#^

^†^Definition for burst sizes are: ${\bar{b}}_{m}=\frac{{k}_{m}}{{\gamma }_{g}+{k}_{g}}$, ${\bar{b}}_{p0}=\frac{{k}_{p}}{{\gamma }_{m}+{\gamma }_{p}}$, ${\bar{b}}_{p}=\frac{{k}_{p}}{{\gamma }_{m}}$.

^‡^The mean and variance of mRNA production with mRNA burst are the mean and variance of the burst events of protein in the case of both bursts.

^*^Definitions for the fractions are: ${F}_{0}=\frac{{\gamma }_{g}({\gamma }_{g}+{\gamma }_{m}+{\gamma }_{p}+{k}_{g})}{({\gamma }_{g}+{\gamma }_{m}+{k}_{g})({\gamma }_{g}+{\gamma }_{p}+{k}_{g})}$, ${F}_{1}=\frac{{\gamma }_{g}}{({\gamma }_{g}+{\gamma }_{m}+{k}_{g})}$, ${F}_{2}=\frac{{\gamma }_{g}}{({\gamma }_{g}+{\gamma }_{p}+{k}_{g})}$.

^§^With the conditions of ${\gamma }_{g}\gg {\gamma }_{m}$ and ${\gamma }_{g}\gg {k}_{g}$, ${F}_{1}$ replaced by $1$ with mRNA burst.

^¶^With the conditions of ${\gamma }_{g}\gg {\gamma }_{m}$ and ${\gamma }_{g}\gg {k}_{g}$, ${F}_{0}$ replaced by $1$ with mRNA burst.

^||^With the conditions of ${\gamma }_{g}\gg {\gamma }_{m}$ and ${\gamma }_{g}\gg {k}_{g}$, ${F}_{0}$ replaced by $1$ with both bursts; with ${\gamma }_{m}\gg {\gamma }_{p}$, ${\bar{b}}_{p0}$ replaced by ${\bar{b}}_{p}$.

^#^With the conditions of ${\gamma }_{m}\gg {\gamma }_{p}$, ${F}_{0}$ replaced by ${F}_{2}$ with protein burst; and with ${\gamma }_{m}\gg {\gamma }_{p}$, ${\bar{b}}_{p0}$ replaced by ${\bar{b}}_{p}$.

#### Neither mRNA nor protein in bursts

For the case that *γ*_*g*_ and *γ*_*m*_ are close to *γ*_*p*_, simulations with the Gillespie algorithm or the *τ*-leaping algorithm^35^ would work well. The problems of inefficient simulation and poor statistics of rare events due to greatly different reaction rates do not exist in this case. Because mRNA and protein production are not in bursts, all three species in equations (1) to (3) need to be tracked in the simulation to fully account for intrinsic noise of gene expression. Simulation with the Gillespie algorithm has no imposed approximation, and thus the steady-state variance it produces is close to LNA in equation (26). Therefore, the lower left region of Fig. 2b indicates zero error.

## Results

### Single gene expression

The gene expression model as described in equations (1) to (3) is tested to see how the one-component burst Langevin equation in equation (31) can be used to replace a three-component model. We use the Gillespie algorithm to simulate the model as in equations (1) to (3) to obtain the exact numerical simulation results. We compared the normalized error of a protein’s mean $(\bar{p})$ in the steady state from the burst Langevin simulation to that from the Gillespie simulation.

There are six parameters in the model as in equations (1) to (3). We first chose the unit for time as 1/*γ*_*p*_. In other words, the protein degradation rate was set to 1. We further set *γ*_*m*_ = 10 and *γ*_*g*_ = 100, for a fast degradation rate in mRNA and an even faster DNA deactivation rate, respectively. This is at the margin of treating both mRNA and protein production with bursts (equation (32)), where the largest error (<6.5%) could be produced, as shown in the upper right region of Fig. 2b. To test the applicable range of the burst Langevin simulation, we scanned the gene activation constant (*k*_*g*_ = 1 − 100), which covers the mRNA burst frequency value of 5 to 45 as observed in the experiment^22^. The other parameter we scanned is the protein burst size $({\bar{b}}_{p}=1-100)$, or equivalently protein production rate (*k*_*p*_ = 10 − 1000), which also covers the values observed in the experiment^28^. We first fixed the parameter *k*_*m*_ as 100, which approximately yields ${\bar{b}}_{m}=1$ by equation (16), similar to the value generally observed in bacteria^28^. The average amount of protein in the steady state from the parameter set we scanned can be calculated as

39 $$\bar{p}=\frac{{k}_{g}}{{k}_{g}+{\gamma }_{g}}\frac{{k}_{m}}{{\gamma }_{m}}\frac{{k}_{p}}{{\gamma }_{p}}=\frac{{k}_{g}}{{k}_{g}+100}\frac{100}{10}\frac{{k}_{p}}{1},$$

which covers $0.9 < \bar{p}\le 5000$, the range of observed protein copy number in an *E. coli* cell^28^.

**Figure 3:**
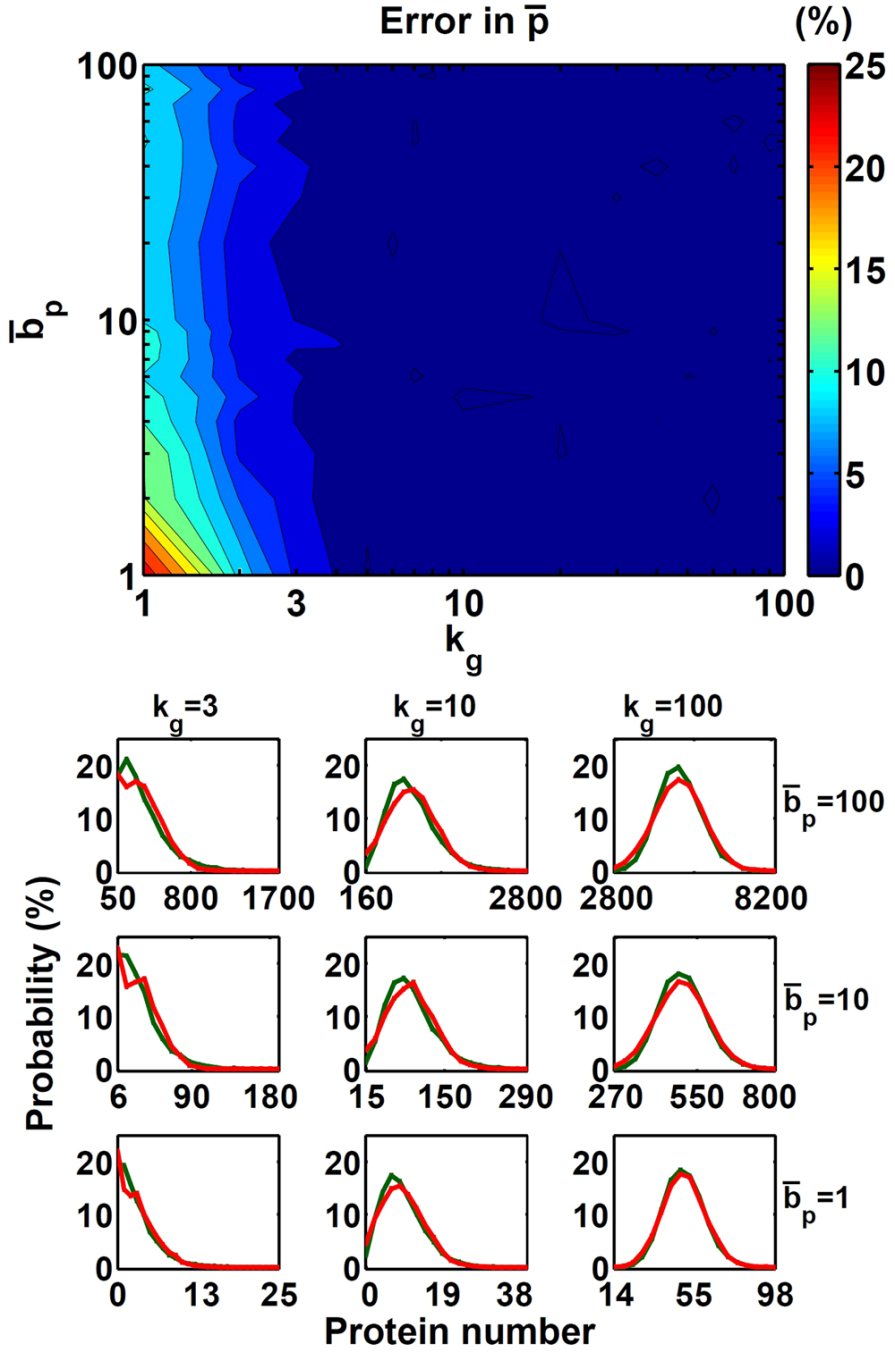
Comparison of $\bar{p}$ and steady-state distributions with the burst Langevin simulation and Gillespie simulation. Shown in (a) are the $\bar{p}$ difference (in %) with the burst Langevin simulation and Gillespie simulation and in (b) steady-state distributions with the burst Langevin simulation (red) and Gillespie simulation (green) with different gene activation rates *k*_*g*_ = 3,10,100 and burst size ${\bar{b}}_{p}=\mathrm{1,10,100}$. Statistics were taken at the steady state of 10,000 independent points for the model as defined in equations (1) to (3) with parameters *k*_*m*_ = 100, *γ*_*g*_ = 100, *γ*_*m*_ = 10 and *γ*_*p*_ = 1.

We compared the average protein number $(\bar{p})$ of the steady-state distribution from the burst Langevin simulation to that from the Gillespie simulation and shown in Fig. 3a. When *k*_*g*_ ≥ 3 and ${\bar{b}}_{p}\ge 1$, corresponding to $\bar{p}\ge 3$, our algorithm’s error is <5%. The largest error of $\bar{p}$ is found at the lower left corner, which is caused by the Gaussian function in the Langevin simulation deviating from the Poisson distribution. Such deviation affects all kinds of Langevin simulations, including our burst Langevin scheme.

Figure 3b compares the protein’s steady-state distribution with the burst Langevin simulation and Gillespie simulation. The burst Langevin simulation can reproduce the distributions with different combinations of burst frequency (*k*_*g*_) and burst size (${\bar{b}}_{p}$).The normalized error in standard deviation for these cases ranges from −13% to 14% (details included in the supplementary information). Although all the steady-state distributions have some error in *σ*_*p*,*ss*_, they are sufficiently good for further applications. We further analyzed the sources of such error and discussed them in the supplementary information for interested readers.

**Figure 4:**
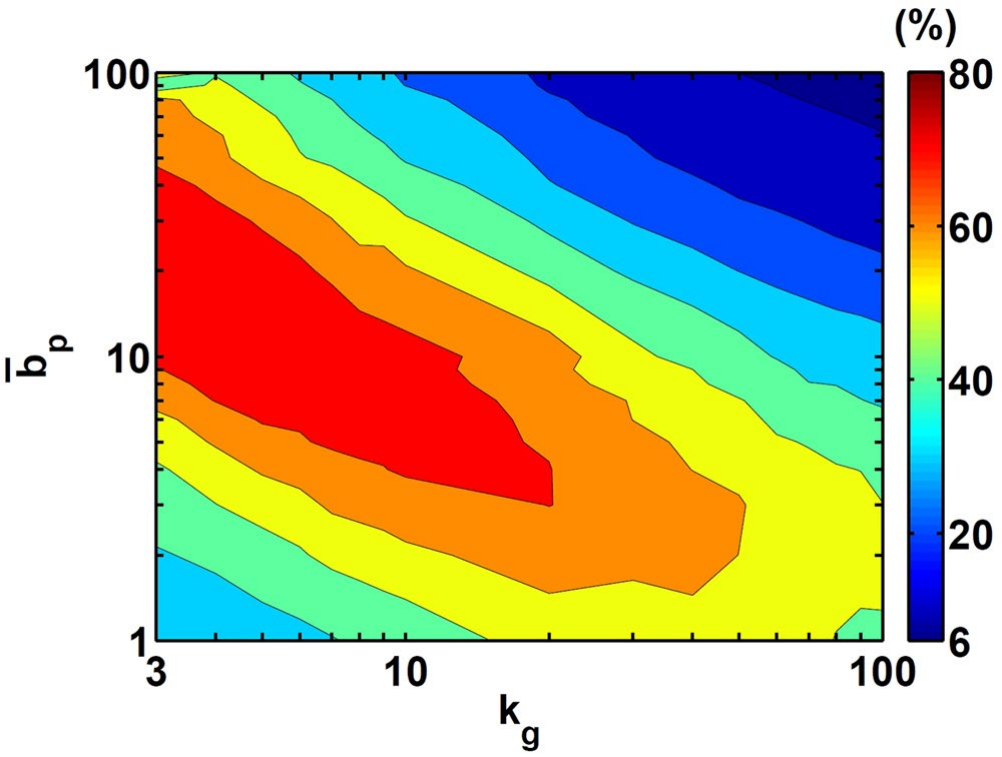
Comparison of simulation time with the burst Langevin simulation and Gillespie simulation. Shown are the percentage of computer time used by the burst Langevin simulation compared to that by the Gillespie simulation for different *k*_*g*_ and burst size ${\bar{b}}_{p}$.

Figure 4 compares the computational time percentage with the burst Langevin simulation to that with the Gillespie simulation. The burst Langevin simulation always uses less time than the corresponding Gillespie simulation. When particle number is ≤100, the Gillespie simulation is already efficient; thus, the time usage with the burst Langevin simulation is 40% to 80% of that with the Gillespie simulation. However, when the particle number is large, the burst Langevin uses only <10% simulation time as compared with the Gillespie simulation. Therefore, the burst Langevin simulation is efficient.

**Figure 5:**
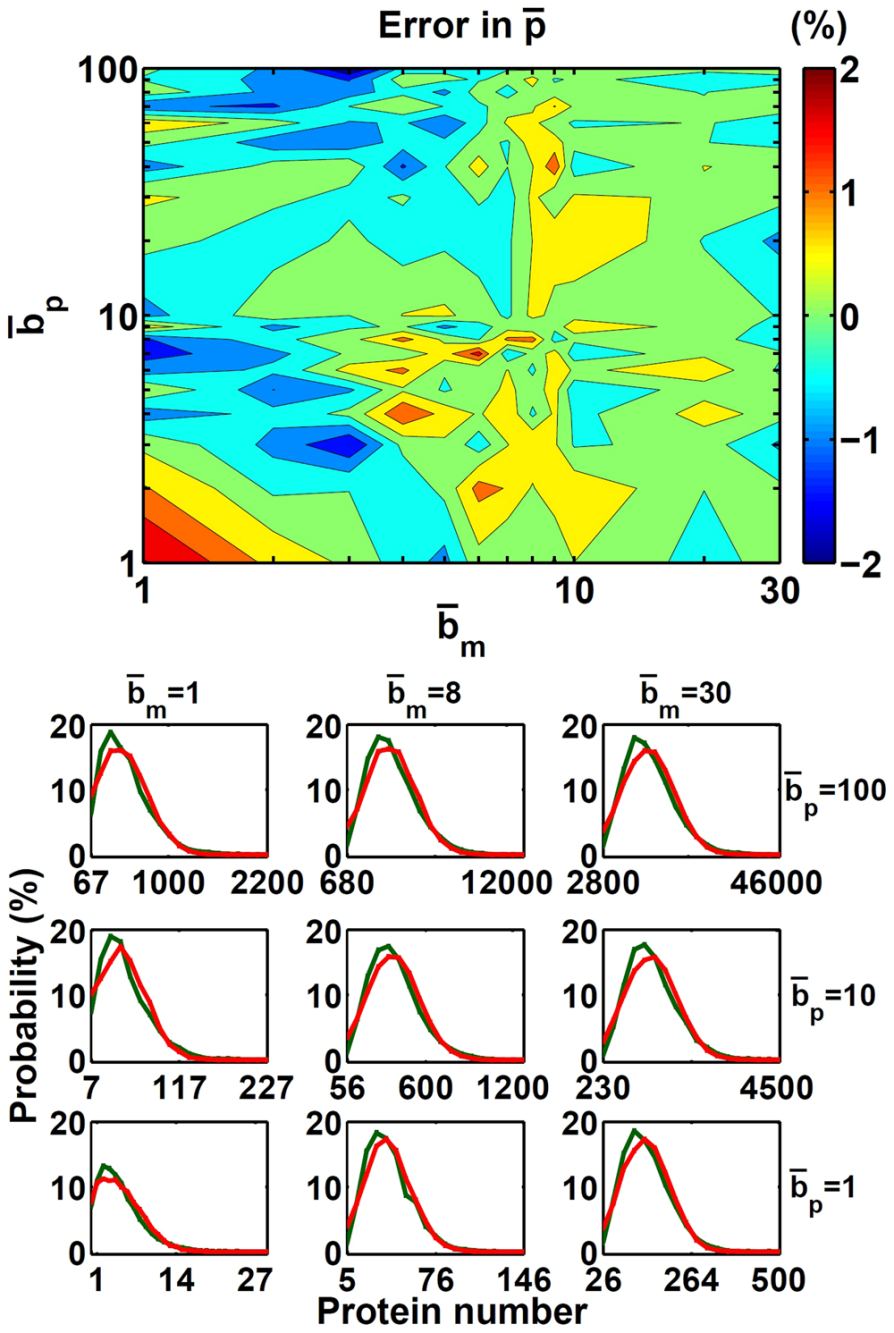
Comparison between the burst Langevin simulation and Gillespie simulation with different ${\bar{b}}_{m}$ and ${\bar{b}}_{p}$. Shown in (a) are the $\bar{p}$ difference (in %) with the burst Langevin simulation and Gillespie simulation and in (b) steady-state distributions with the burst Langevin simulation (red) and Gillespie simulation (green) with different ${\bar{b}}_{m}=\mathrm{1,8,30}$ and ${\bar{b}}_{p}=\mathrm{1,10,100}$. *k*_*p*_ and *k*_*m*_ were determined by equations (5) and (16) with given ${\bar{b}}_{p}$ and ${\bar{b}}_{m}$, respectively, with the other parameters *k*_*g*_ = 5, *γ*_*g*_ = 100, *γ*_*m*_ = 10 and *γ*_*p*_ = 1.

We also checked the accuracy of the burst Langevin simulation comparing to the Gillespie simulation by varying mRNA burst size. In this test, with a fixed *k*_*g*_ = 5, we scanned the other parameter pair: mRNA mean burst size, ${\bar{b}}_{m}=1-30$ and protein mean burst size, ${\bar{b}}_{p}=1-100$. The parameter ${\bar{b}}_{m}=1-30$ corresponds to the mRNA burst size observed in mammalian cells^22^. The parameter region tested corresponds to $\bar{p}=4-\mathrm{15,000}$. As shown in Fig. 5a, the errors in $\bar{p}$ are within ±2%. The steady-state distributions between two methods shows a good agreement (Fig. 5b). Comparison of the standard deviation in the steady state (from −8% to 2.5%) is included in the supplementary information. These results indicate that our burst Langevin algorithm is applicable for a wide range of biological systems.

### Burst Langevin for non-linear regulation

We further tested a gene’s expression under regulation to show how steady-state distribution errors of the upstream can affect the downstream mean number, especially with a non-linear regulation. Here we chose repressing regulation as an example. We use the gene expression model shown in equations (1) to (3) as an upstream protein, *p*_1_ with varying ${\bar{b}}_{m1}$ and ${\bar{b}}_{p1}$ (as in Fig. 5a). The downstream gene’s transcription is repressed by *p*_1_ through the Hill function with threshold (*K*) as in the following equations:

40 $$\frac{d{p}_{1}}{dt}={k}_{g1}{\bar{b}}_{m1}{\bar{b}}_{p1}-{\gamma }_{p1}\,{p}_{1},$$

41 $$\frac{d{p}_{2}}{dt}={k}_{g2}({\bar{b}}_{m2}\frac{{K}^{{n}_{H}}}{{K}^{{n}_{H}}+{p}_{1}^{{n}_{H}}}+{k}_{l}){\bar{b}}_{p2}-{\gamma }_{p2}\,{p}_{2}.$$

We compared the difference in ${\bar{p}}_{2}$ between the burst Langevin simulation and Gillespie simulation. The simulation result for an activation regulation with negative *n*_*H*_ (equivalent to a positive regulation) can be found in the supplementary information. We introduced *k*_*l*_ for *p*_2_’s possible leaking of mRNA, so that the expression of an repressed gene may remain at a low level but not zero^36^. In this way, a basal production for *p*_2_ is introduced, and thus, the problem of the Langevin simulation with very low *p*_2_ can be mostly avoided.

**Figure 6:**
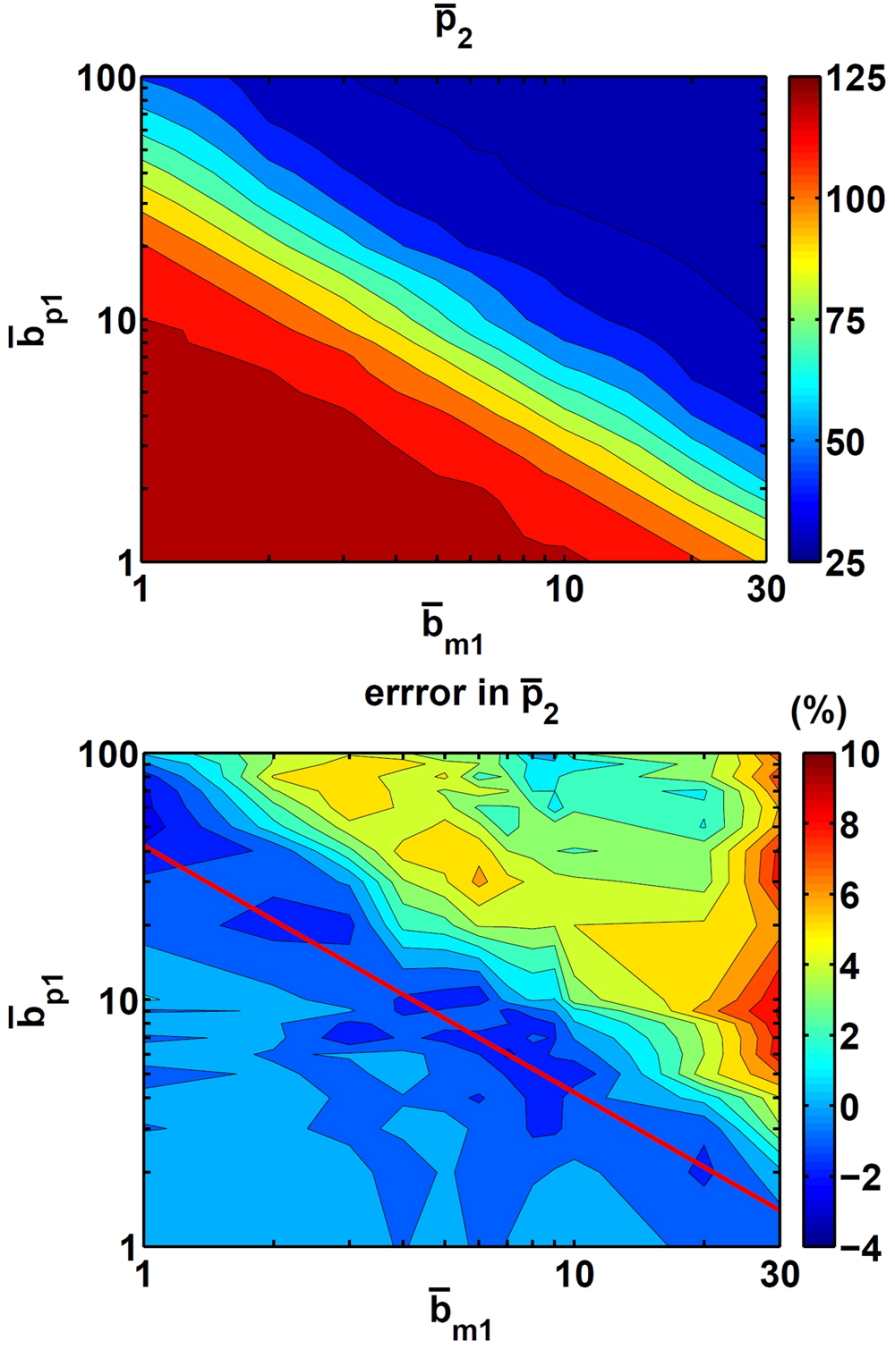
A test for simulation error of gene expression under non-linear repressive regulation. Shown in (a) is the steady-state ${\bar{p}}_{2}$ with the burst Langevin simulation and in (b) the error in ${\bar{p}}_{2}$ from the burst Langevin simulation comparing to that from the Gillespie simulation. Here the *p*_1_’s burst frequency, ${\bar{b}}_{m1}$, and burst size, ${\bar{b}}_{p1}$, are varied over a range. Other parameters for *p*_1_ are *k*_*g*1_ = 5, *γ*_*g*1_ = 100, *γ*_*m*1_ = 10 and *γ*_*p*1_ = 1. For *p*_2_, the parameters are *k*_*g*2_ = 5, *γ*_*g*2_ = 100, *k*_*m*2_ = 200, *k*_*l*_ = 60, *γ*_*m*2_ = 10, *k*_*p*2_ = 100 and *γ*_*p*2_ = 1, *K* = 200 and *n*_*H*_ = 3. The red line in (b) corresponds to ${\bar{p}}_{1}=K$.

In the lower-left corner of Fig. 6a, the downstream gene expression level is ${\bar{p}}_{2}=125$, which means that *p*_2_ is fully activated and there are only a few *p*_1_. With increasing *p*_1_, *p*_2_ is reduced to ${\bar{p}}_{2}=25$ as seen in the upper-right corner of Fig. 6a. The errors in ${\bar{p}}_{2}$ in Fig. 6b are from −4% to 8%. The red line in Fig. 6b indicates ${\bar{p}}_{1}=K$, the threshold value of the repression. Within the region close to the threshold, the production of *p*_2_ is sensitive to fluctuations in *p*_1_. However, even in this region nearby, the error at most is only −4%. Therefore, even with a non-linear regulation in this system, the burst Langevin simulation can produce accurate results.

In Fig. 6b, the largest error in ${\bar{p}}_{2}$ is about 8%, found with ${\bar{b}}_{m1}=30$ and ${\bar{b}}_{p1}\ge 5$. In this region, *p*_1_’s copy number is high, and its number fluctuation is also high with such large burst-size pairs. *p*_2_ is fully repressed by high *p*_1_ number and kept at its basal expression level, ${\bar{p}}_{2}=25$. Here the 8% error comes from a two- to three-particle difference in ${\bar{p}}_{2}$, and such error is quite acceptable in stochastic simulations.

In the region that we scanned, *p*_1_’s *σ*_*p*1,*ss*_ error is from −8.5% to 2.5%, which mainly follows the value of ${\bar{b}}_{m1}$ (as shown in supplementary information). Such error may propagate through the regulation and cause error in ${\bar{p}}_{2}$. However, as seen in Fig. 6b, the error in ${\bar{p}}_{2}$ has only a mild correlation with increasing ${\bar{b}}_{m1}$ alone. The overall trend of increasing error roughly follows inversely with increasing *p*_2_ from the down-left corner to up-right corner in Fig. 6b, and thus, the errors in the standard deviation of the upstream do not affect the quality of the downstream.

### Dynamics of average protein number

**Figure 7:**
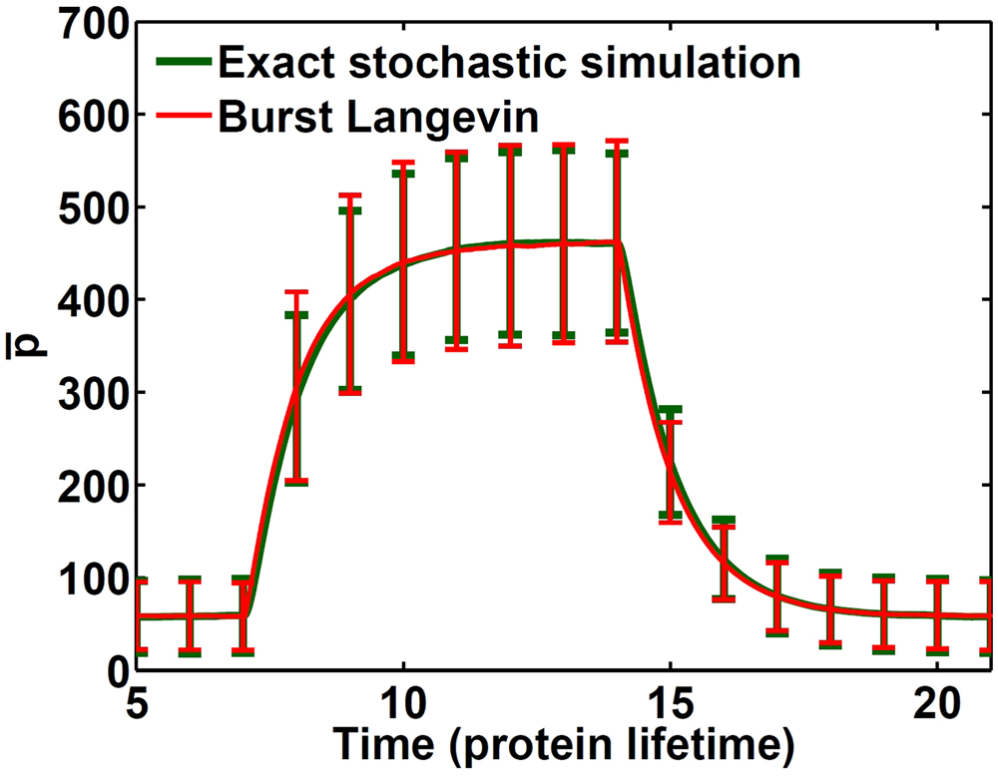
Comparison of different algorithms for genetic switching dynamics. Shown are average protein numbers with the standard deviation of the distribution at different times from 10,000 independent stochastic trajectories with the burst Langevin algorithm (red) and Gillespie simulation (green) for the model defined in equations (1) to (3) with parameters *k*_*g*_ = 30 for *t* = 7 to 14; otherwise *k*_*g*_ = 3 and other parameters *γ*_*g*_ = 100, *k*_*m*_ = 200, *γ*_*m*_ = 10, *k*_*p*_ = 100 and *γ*_*p*_ = 1.

Besides steady-state behaviors, we demonstrate the accuracy of the burst Langevin simulation in dynamics. In Fig. 7, we include the mean protein number dynamics with the burst Langevin simulation and Gillespie simulation. In this model, the gene is activated at time $t=7$ by setting *k*_*g*_ = 30 and deactivated at *t* = 14 by setting *k*_*g*_ = 3. From Fig. 7, we can see that our simulation algorithm can produce reasonably accurate dynamics in the mean and standard deviation as compared with the exact Gillespie simulation. Only a small deviation can be found in the standard deviation. In this test, we selected ${\bar{b}}_{m}=2$ and ${\bar{b}}_{p}=10$. However, similar results with reasonable dynamics are obtained by varying combinations of ${\bar{b}}_{m}$ and ${\bar{b}}_{p}$ (supplementary information).

## Discussion

In this work, we have developed a Langevin equation that can account for the noise arising from gene expression bursts. We found a large range of parameters with which our burst Langevin simulation can well reproduce the statistics comparing to the Gillespie algorithm, and it covers the protein expression level for more than 4 orders of magnitude. For the case of mRNA (protein) burst production, the deactivation (degradation) rates of the gene (mRNA) should be 10 times faster than that of mRNA (protein). The burst Langevin equation has the flexibility to include only mRNA or protein burst or both bursts. In addition, the gene activation rate constant (*k*_*g*_) has multiple effects in the mean and variance of the production distribution; thus, it is a critical parameter in the accuracy of the burst Langevin simulation. When *k*_*g*_ ≥ 3, which leads to $\bar{p}\ge 3$, the burst Langevin simulation can produce an accurate steady-state mean and standard deviation as compared with the Gillespie simulation. Furthermore, the burst Langevin simulation can produce accurate dynamics of genetic switching and genes under non-linear regulation. Therefore, the burst Langevin equation is applicable for a wide range of genetic regulation network.

To fully consider all intrinsic noises of a gene, with the Gillespie simulation, all of the three components including the gene state, mRNA and protein are simulated with a total of six reaction channels. However, with the burst Langevin simulation, with both mRNA and protein bursts, the model can be reduced to only protein with two reaction channels. Thus, the burst Langevin simulation uses less computational time and memory than the Gillespie simulation. Therefore, our algorithm is an efficient stochastic simulation method.

Besides efficiency, the Langevin equation allows for easily dissecting the contribution of noise from different sources, because the Gaussian random number for each reaction channel can be easily set as zero. Therefore, the burst Langevin simulation can be used to analyze the dynamics of gene expression noises propagating through the regulation network^8,11^. Moreover, one can introduce a desirable scaling parameter to the noise strength in the Langevin equation for mimicking other possible sources. Therefore, one can reproduce the noises close to that from the Gillespie simulation or that observed in various biological systems.

With the variance expression in equation (11), our burst Langevin equation can be flexible to include various cellular factors. With the assumption that burst events and burst sizes are determined by independent processes, we use two different distributions to estimate the variance of burst production in the burst Langevin equation. For the cases of mRNA or protein burst alone, we use the Poisson distribution for burst events and geometric distribution for burst sizes, whereas for the case of both bursts, the protein burst event’s variance is enhanced by the mRNA burst, and the overall variance is obtained by the same expression in equation (11). In general, gene expression in the model (equations (1) to (3)) may be influenced by other factors in the cell^37^, such as chromatin template and promoter structure^38,39^, which leads to different mRNA and protein production distributions other than Poisson or geometric distributions^40^. Also, post-translational modification introduces an additional step after protein production, which can modify the overall protein production rate, *k*_*p*_. Thus, protein burst size ${\bar{b}}_{p}$ and variance ${\sigma }_{bp}^{2}$ may also be modified from the geometric distribution. For a more detailed model^40^, if burst event and size are determined independently, their distributions can be introduced in equation (11) to estimate consequent burst production variance. Therefore, the burst Langevin equation in equation (31) can be modified accordingly to include other factors or more detailed steps in the gene expression model.

Different steps in gene expression are implemented by different molecular machineries. A gene is activated by chromatin remodeling^4,21^, whereas mRNA is produced by RNA polymerase and protein is produced by ribosome. There is no machinery competition between different steps. Therefore, we assumed that different steps in gene expression are independent processes and derived the variance of burst production. However, under different physiological conditions in bacteria^41,42^, negative correlations are reported between transcription and translation. And thus, regulation or competition for resources may exist between transcription and translation. Yet, once a gene is expressing, protein requires more biomass than transcripts. Protein synthesis also consumes most of the energy, but other processes in gene expression consume a non-relevant amount (<10%) of energy^43,44^. Therefore, energy competition may only be possible in some extreme conditions and assuming different steps in gene expression as independence processes is valid.

## Methods

### Burst Langevin Simulation Settings

#### *τ* selection for burst events

In propagating the Langevin equation, the Euler-Maruyama scheme^45^ is technically rather simple to implement. While using this scheme, we need to select a proper step size *τ*, such that all reactant’s expected changes are within a small proportion, *ε*. For a general biological model, besides the protein’s burst production and degradation in equation (31), the protein may involve other *j*th reaction with reaction propensity *a*_*j*_ and number change *ν*_*pj*_:

42 $$\begin{array}{rcl}p(t+\tau ) & = & p(t)+({k}_{g}{\bar{b}}_{m}{\bar{b}}_{p}\tau +{({k}_{g}{\bar{b}}_{m}{\bar{b}}_{p}\tau (2{\bar{b}}_{m}{\bar{b}}_{p}+2{\bar{b}}_{p}+1))}^{\mathrm{1/2}}{{\mathscr{N}}}_{1}\mathrm{(0,}\,\mathrm{1)})\\  &  & -\,({\gamma }_{p}p\tau +{({\gamma }_{p}p\tau )}^{\mathrm{1/2}}{{\mathscr{N}}}_{2}\mathrm{(0,}\,\mathrm{1)})\\  &  & +\,\sum _{j}({\nu }_{pj}{a}_{j}\tau +{({\nu }_{pj}{a}_{j}\tau )}^{\mathrm{1/2}}{{\mathscr{N}}}_{j}\mathrm{(0,}\,\mathrm{1)})\mathrm{.}\end{array}$$

Following the *τ*-leaping scheme^35^, the step size *τ* is determined by:

43 $$\tau =\mathop{\min }\limits_{i}\{\frac{\max \{\varepsilon p,1\}}{|{k}_{g}{\bar{b}}_{m}{\bar{b}}_{p}+(-1){\gamma }_{p}p+\sum _{j}{\nu }_{pj}{a}_{j}|},\frac{\max \,{\{\varepsilon p,1\}}^{2}}{{k}_{g}{\bar{b}}_{m}{\bar{b}}_{p}+{(-1)}^{2}{\gamma }_{p}p+\sum _{j}{\nu }_{pj}^{2}{a}_{j}},{\tau }_{i\ne p}\},$$

where the first denominator multiplied by *τ* is *p*’s expected change in *τ* and the second denominator multiplied by *τ* is the variance of expected change. The *τ* is selected as the minimum *τ*_*i*_ among all reacting species *i*, including *p*. The setting in the numerator is for the efficiency of simulation. When the amount of protein is large, the term *εp* in the numerator includes as many reactions as possible in *τ* and accelerates the simulation being faster than Gillespie algorithm. When there are only a few proteins, with the second term, 1 in the numerator, the *τ* is large enough for some reactions such that reactant numbers are changed at least by one particle. In the situation of few particles, the *τ*-leaping scheme is close to the Gillespie algorithm, which tracks every reaction.

There are some reactions whose propensity changes drastically even with one reaction event. These are classified as critical reactions in the system^35^. Examples are the switching steps between the two gene states in equation (34) or the protein degradation reaction with protein number <10, where the number 10 is suggested in the literature^35^. Besides the *τ* selected from equation (43), when there are critical reactions in the current state, another *τ*_*c*_ is randomly selected from an exponential distribution function with ${\bar{\tau }}_{c}$, which is the average time for one critical reaction event. The ${\bar{\tau }}_{c}$ is defined as the reciprocal of the sum of all critical reaction propensities. If *τ*_*c*_ is smaller than the *τ* from equation (43), the system is propagated with *τ*_*c*_ with one critical reaction event. Otherwise, the system is propagated with *τ* without any critical reactions.

However, in the Langevin simulation with bursts, a large-size burst causes an additional problem in the *τ*-leaping scheme^35^ when the amount of protein is low. In equation (43), large ${k}_{g}{\bar{b}}_{m}{\bar{b}}_{p}$ value in the denominator leads to a very small *τ*, and only one protein is produced. And such small *τ* will be selected consecutively for a complete burst event. No reactions occur in such small *τ* other than one protein produced, and thus, the simulation time is wasted. To overcome this situation, we reformulated equation (43) such that one burst event is allowed in one *τ* step. With this modification in mind, we consider two different *τ*’s, one estimated from the burst production and the other $({\tau }_{pj\in nb})$ from other non-burst reactions (including protein degradation) following the original scheme as in equation (43). The smaller *τ* between the two is then chosen. Therefore, our *τ* selection scheme is modified as:

44 $$\tau =\mathop{\min }\limits_{i}\{\mathop{\min }\limits_{i=p}\{\max \{\frac{\varepsilon p}{{k}_{g}{\bar{b}}_{m}{\bar{b}}_{p}},\frac{{\bar{b}}_{p}}{{k}_{g}{\bar{b}}_{m}{\bar{b}}_{p}}\},{\tau }_{pj\in nb}\},{\tau }_{i\ne p}\}.$$

The first fraction is still the same as that from equation (43) for only burst production. When *εp* ≤ 1, we multiply the original selection of $\tau =\mathrm{1/}{k}_{g}{\bar{b}}_{m}{\bar{b}}_{p}$ by ${\bar{b}}_{p}$ to include a whole burst event. Further detailed models included effects of chromatin template and promoter structure^38,39^, which change the waiting time distribution for next burst event. And thus, the term $\tau =\mathrm{1/}{k}_{g}{\bar{b}}_{m}$, which is derived from an exponential distribution, needs to be modified accordingly if the chromatin structural change is considered. Other detailed considerations are included in the supplementary information accompanying this work.

With a selected *τ*, we can determine the protein’s burst production number and degradation number according to equation(31). When *p*(*t*) is small, a randomly selected degradation number may be so large that negative *p*(*t* + *τ*) is obtained. If *p*(*t* + *τ*) becomes negative, we take half of the originally selected *τ*, such that particle changes become small, and repeat this procedure if necessary, until *p*(*t* + *τ*) ≥ 0. Such half-*τ* scheme is suggested in the work of Cao *et al*.^35^.

#### Removing negative production

**Figure 8:**
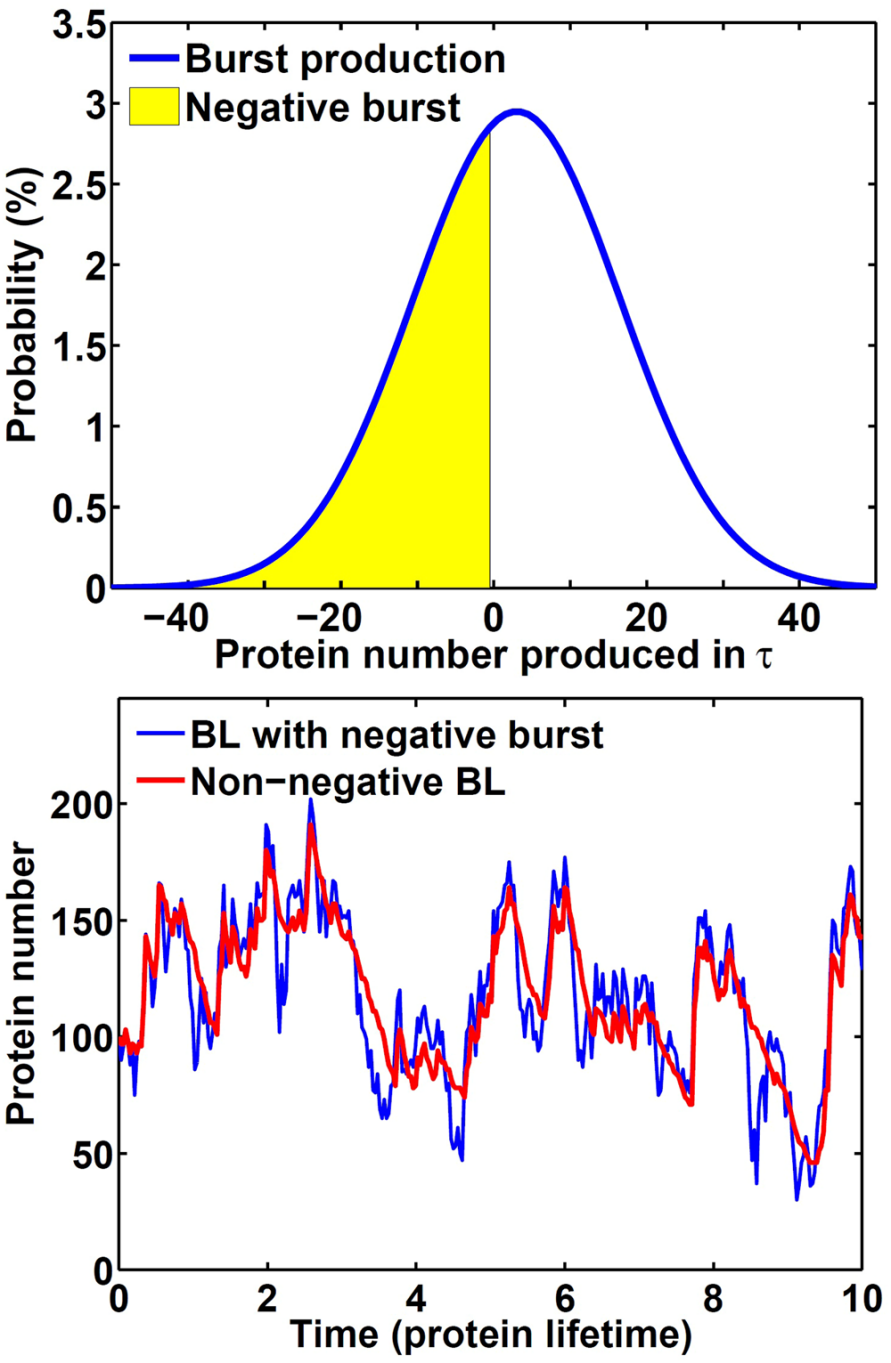
A representative burst production distribution and the effect of negative burst on a protein’s fluctuation. Shown in (a) is a typical burst production distribution as defined in equation (31) with *τ* = 0.03 and colored area as the negative production and in (b) are two stochastic trajectories from the algorithm following equation (31), with (blue) and removing (red) negative burst production. Parameters are *k*_*g*_ = 5, *γ*_*g*_ = 95, *k*_*m*_ = 200, *γ*_*m*_ = 10, *k*_*p*_ = 100 and *γ*_*p*_ = 1, corresponding to $\bar{p}=100$ with ${\bar{b}}_{m}{\bar{b}}_{p}=20$.

The protein’s production number in each *τ* is calculated by the second term in the right-hand side of equation (31) and then rounded to the nearest integers. Shown in Fig. 8a is the Gaussian distribution we used to approximate the burst production. With *τ* = 0.03, the mean production number is 3 and the standard deviation is about 13.5. From such Gaussian distribution, there is a nearly 40% chance to obtain a negative random number, which is then rounded to a negative production number when it is ≤−0.5. Even with a high expression level, with *k*_*g*_ = 100 and ${\bar{b}}_{p}=100$, the negative part still can be >15% of burst production. A more complete profile for the negative burst percentages is included in the supplementary information. Such negative production also reduces the protein number as the degradation process and leads to many sudden drops in the blue trajectory in Fig. 8b. Negative production is an artifact due to the Gaussian distribution used in the Langevin simulation.

We used a rather simple approach to remove such negative productions and keep the mean of the distribution at the same time. When a negative production number is selected, the negative number is temporarily stored for accumulation with the next production. The production in the current time step is zero, as the silent moment between two bursts. Only when the accumulated protein number becomes positive is there a burst with such a positive number, and the protein number is increased by production. As seen in Fig. 8b, the unrealistic sudden drop is removed in the red trajectory. We note that the red trajectory has a similar shape due to the rapid production and slow degradation as in Fig. 1b.

### Data Availability

All data generated or analysed during this study are included in this published article (and its supplementary information file).

## Electronic supplementary material

Supplementary information
