# Supplementary material for: Efficient and flexible implementation of Langevin simulation for gene burst production
Source: Sci Rep. 2017 Dec 4;7:16851. doi: 10.1038/s41598-017-16835-y (PMC5715166; doi:10.1038/s41598-017-16835-y)
Supplement: Supplementary file 1 — Supplementary information [file 41598_2017_16835_MOESM1_ESM.pdf]

Supplementary information for "Efficient and flexible implementation of Langevin simulation for gene burst production"

Ching-Cher Sanders Yan,[1] Surendhar Reddy Chepyala,[1,2,3] Chao-Ming Yen,[4] and Chao-Ping Hsu[1,5,a)]

[1)] *Institute of Chemistry, Academia Sinica, 128, Section 2, Academia Road, Nankang, Taipei, 115, Taiwan*

[2)] *Bioinformatics Program, Taiwan International Graduate Program, Institute of Information Science, Academia Sinica, 128, Section 2, Academia Road, Nankang, Taipei, 115, Taiwan.*

[3)] *Institute of Biomedical Informatics, National Yang-Ming University, Taipei, 112, Taiwan*

[4)] *Institute of Biochemical Sciences, College of Life Science, National Taiwan University, 1, Section 4, Roosevelt Road, Taipei, 106, Taiwan*

[5)] *Genome and Systems Biology Degree Program, National Taiwan University, 1, Section 4, Roosevelt Road, Taipei, 106, Taiwan*

(Dated: 15 November 2017)

[a)] Corresponding author: cherri@chem.sinica.edu.tw

CONTENTS

I. Stochastic descriptions for gene expression 3

A. Chemical master equation 3

B. Linear noise approximation 4

C. Stochastic simulation following Gillespie algorithm 5

D. Chemical Langevin equation 6

II. Steady-state variance from burst Langevin equation 7

III. The modified τ-leaping scheme for burst production 8

IV. Supplementary results 11

A. standard deviation comparison among different methods 11

B. k_g and $\bar{b}_m$ are more critical than $\bar{b}_p$ for the accuracy of $\sigma_{p,ss}$ 16

C. Significantly small k_g and γ_g leading to protein number's bimodal distributions 16

D. Burst Langevin for non-linear activation 17

E. Genetic switching dynamics with different parameter sets 17

References 18

In this supplementary information, first section includes a brief review of four different stochastic descriptions for the gene expression. First, a model with protein burst[1] is taken as an example to introduce the chemical master equation (CME). The CME is a complete description for the stochastic dynamics[2–4]. However, the solution is intractable for a large system with a huge number of possible states. In such situation, the second stochastic description we introduced, the linear noise approximation (LNA)[5,6] is usually considered. For all components in the CME, the LNA can yield analytical expressions for their steady-state variance. Thereby, LNA is used to obtain the steady-state variance expressions for the gene state, mRNA and protein in the gene expression model (Eqs. (1) to (3)) of the main text. Gillespie algorithm is the third stochastic description that we introduced. This algorithm renders the CME without further approximations, and thus, we took the output from the Gillespie simulations as the standard for further comparisons. As the fourth description, the complete expression of chemical Langevin equations is included for the gene expression. Unlike in the main text, the Langevin equation for the short-lived components are included. In the second section, we derived the steady-state variance directly from the burst Langevin equation. In the third section, we included the detailed considerations for modifying the τ selection scheme in the burst Langevin simulations. Finally, we show additional simulation results supporting the discussions in the main text. At the end of this file, a list of symbols used in this work is included.

I. STOCHASTIC DESCRIPTIONS FOR GENE EXPRESSION

Here we provide a brief review of four stochastic descriptions for the model of gene expression. The focus is on the CME and LNA, which are important and fundamental, though they are not introduced with details in the main text.

A. Chemical master equation

We reviewed the work of Friedman *et al*[1] as an example for using the CME to describe gene expression with protein burst production. Protein burst can be obtained by including

the mRNA fluctuation as follow:

$$\frac{dm}{dt} = k_m - \gamma_m m \tag{S1}$$

$$\frac{dp}{dt} = k_p m - \gamma_p p. \tag{S2}$$

The two equations are the same as Eqs. (2) and (3) in the main text with g fixed as 1. The CME for this model describes the dynamics of probability distribution function, $P(m, p; t)$:

$$\begin{aligned}\frac{\partial}{\partial t}P(m,p;t) &= k_m P(m-1,p;t) - k_m P(m,p;t) \\ &+ \gamma_m m P(m+1,p;t) - \gamma_m m P(m,p;t) \\ &+ k_p m P(m,p-1;t) - k_p m P(m,p;t) \\ &+ \gamma_p p P(m,p+1;t) - \gamma_p p P(m,p;t),\end{aligned} \tag{S3}$$

where the states (m, p) are specified by varying mRNA and protein numbers. Particle numbers are changed through four different reactions. Each reaction contributes two terms for the incoming and outgoing probabilities for states of (m, p). By assuming the amount of protein as a continuous variable, this CME can be solved.[1] From the solution, when proteins are produced in bursts, protein's steady-state variance is proportional to the average burst size fo protein:

$$\sigma^2_{p,ss} \approx \bar{p}\bar{b}_p. \tag{S4}$$

B. Linear noise approximation

The steady-state variance of g, m and p in Eqs. (1) to (3) from the main text can be derived by the LNA. Here we include the calculation steps for this gene expression model. The steady-state variance matrix $\mathbf{V}$ is defined as:

$$\mathbf{V} = \begin{pmatrix} \sigma^2_{g,ss} & \delta g \delta m & \delta g \delta p \\ \delta g \delta m & \sigma^2_{m,ss} & \delta m \delta p \\ \delta g \delta p & \delta m \delta p & \sigma^2_{p,ss} \end{pmatrix}. \tag{S5}$$

It can be solved from the following equation:

$$\mathbf{AV} + \mathbf{VA}^T + \mathbf{D} = 0, \tag{S6}$$

with matrix $\mathbf{A}$, dynamic Jacobian, as:

$$\mathbf{A} = \begin{pmatrix} -k_g - \gamma_g & 0 & 0 \\ k_m & -\gamma_m & 0 \\ 0 & k_p & -\gamma_p \end{pmatrix}, \tag{S7}$$

and diffusion matrix $\mathbf{D}$ as:

$$\mathbf{D} = \begin{pmatrix} k_g(1-g) + \gamma_g g & 0 & 0 \\ 0 & k_m + \gamma_m m & 0 \\ 0 & 0 & k_p m + \gamma_p \end{pmatrix}. \tag{S8}$$

The general definition of each term in $\mathbf{A}$ is

$$A_{ik} = \sum_j \nu_{ij} \left.\frac{\partial a_j}{\partial x_k}\right|_{x_{k,ss}}, \tag{S9}$$

and the definition of each term in $\mathbf{D}$ is

$$D_{ik} = \sum_j \nu_{ij} \nu_{kj} a_j. \tag{S10}$$

These definitions can be found in the quick review of solving CME with LNA in the supplementary material of our previous work[7].

The analytical expressions of the steady-state variances are obtained as:

$$\sigma^2_{g,ss} = \frac{k_g \gamma_g}{(k_g + \gamma_g)^2} = \bar{g}\,(1 - \bar{g}) \tag{S11}$$

$$\sigma^2_{m,ss} = \bar{m} \left(\frac{\gamma_g k_m}{(k_g + \gamma_g)(k_g + \gamma_g + \gamma_m)} + 1 \right) \tag{S12}$$

$$\sigma^2_{p,ss} = \bar{p} \left(\frac{\gamma_g (\gamma_g + \gamma_m + \gamma_p + k_g)}{(\gamma_g + \gamma_m + k_g)(\gamma_g + \gamma_p + k_g)} \frac{k_m}{k_g + \gamma_g} \frac{k_p}{\gamma_m + \gamma_p} + \frac{k_p}{\gamma_m + \gamma_p} + 1 \right). \tag{S13}$$

Since the gene expression model is linear, the variance expression from the LNA had been shown to be exact.[8] Therefore, we consider Eq. (S13), the same as Eq. (26) in main text, as the exact analytical variance expression for the protein.

C. Stochastic simulation following Gillespie algorithm

$P(g, m, p; t)$ of CME can be numerically obtained from the trajectories produced with the Gillespie algorithm[9,10]. In the simulation, total reaction probability is calculated as:

$$a_0 = \sum_j a_j. \tag{S14}$$

The time of the system is randomly propagated by dt according to the expression:

$$dt = \frac{1}{a_0} \ln \left(\frac{1}{1 - r_1} \right), \tag{S15}$$

where r_1 is a random number from a uniform distribution ranged from zero to one. The original expression is $\frac{1}{a_0} \ln \left(\frac{1}{r_1} \right)$. Most random number generators may produce zero, but not producing one. When $r_1 = 0$, there is the numerical problem of $1/0$. And thus, we modify $\frac{1}{r_1}$ to $\frac{1}{1-r_1}$ to avoid such numerical problem. With the propagated time, dt, the jth reaction is also randomly selected according to the second random number, r_2:

$$\sum_{j'=1}^{j} a_{j'} > r_2 a_0, \tag{S16}$$

then the corresponding particle number x_i is updated by ν_{ij}. The discreteness of particle number is included in the Gillespie algorithm.

D. Chemical Langevin equation

To accelerate the stochastic simulation, the system can be propagated with a longer time interval τ with more reaction events than the dt in the Gillespie algorithm with just one reaction event. It is assumed that after a time leap of τ, all reaction propensities do not change significantly. Therefore, the number of events in τ for each reaction can be estimated. Because the reaction propensities are similar, different reactions happen in time interval τ are assumed independent, and they can regarded as Poisson events,[4] with average event number being the same as the corresponding variance. The chemical Langevin equation for the gene expression model can be written as:

$$\begin{aligned}
g(t+\tau) - g(t) &= k_g (1-g)\tau - \gamma_g g \tau \\
m(t+\tau) - m(t) &= \left[k_m g \tau + (k_m g \tau)^{1/2} \mathcal{N}_1(0,1) \right] - \left[\gamma_m m \tau + (\gamma_m m \tau)^{1/2} \mathcal{N}_2(0,1) \right] \\
p(t+\tau) - p(t) &= \left[k_p m \tau + (k_p m \tau)^{1/2} \mathcal{N}_3(0,1) \right] - \left[\gamma_p p \tau + (\gamma_p p \tau)^{1/2} \mathcal{N}_4(0,1) \right].
\end{aligned}$$

As in the main text, the gene in the above equations is assumed as single-copy, and thus, the gene only switches between on and off states, not follow a Poisson distribution.

For the chemical Langevin equation of each reaction, the deterministic propagation part is the same as the original chemical kinetic equation. The fluctuation part of each reaction

is its noise magnitude multiplied with a zero-center, unity-variance Gaussian random variable, $\mathcal{N}_j(0,1)$. However, when the gene is in low expression, the Langevin simulation is not recommended. The reason is that when the average number of reaction event is small, deviation exists between the Gaussian function and the Poisson distribution, even the Gaussian function has the same mean and variance as the Poisson distribution. In addition, when particle number is small, one reaction may change reaction propensities a lot, then, τ may be selected very small. Such selected τ can be smaller than dt from the Gillespie algorithm. Examples are the gene state, $g(t)$, being either 0 or 1, and mRNA being in low copy numbers. For such problems, the τ-leaping scheme were proposed[11] to maintain the accuracy and efficiency of simulations with low copy number particles. On the other hand, the burst Langevin equation we derived in the main text also can bypass the low-copy number problem by skipping simulating gene state or mRNA number.

II. STEADY-STATE VARIANCE FROM BURST LANGEVIN EQUATION

We derived mRNA's steady-state variance $\sigma^2_{m,ss}$ from the burst Langevin equation Eq. (21) in the main text and compare it with LNA's analytical expression in Eq. (S12) (or Eq. (24) in the main text). We write the burst Langevin equation in the differential form and include ξ and η as the zero-centered random variables respectively for production and degradation:

$$\frac{dm}{dt} = k_g\bar{b}_m + \xi - (\gamma_m m + \eta) \tag{S17}$$

$$\langle \xi\xi' \rangle = k_g\bar{b}_m \left(2\bar{b}_m + 1\right) \tag{S18}$$

$$\langle \eta\eta' \rangle = \gamma_m m. \tag{S19}$$

The main production $k_g\bar{b}_m$ is obtained by replacing gk_m in Eq. (2) in main text with $\bar{g} = k_g/\left(k_g + \gamma_g\right)$ and using the definition of $\bar{b}_m = k_m/\left(k_g + \gamma_g\right)$.

Following the same process in the supporting online materials of Pedraza and van Oudenaarden's work,[12] steady-state variance $\sigma^2_{m,ss}$ can be obtained by transforming $m(t)$ to the

Fourier space (variables with hat) first, then squaring, averaging, and transforming back:

$$\delta\hat{m} = \frac{\hat{\xi} + \hat{\eta}}{i\omega + \gamma_m} \tag{S20}$$

$$\left\langle \delta\hat{m}^2 \right\rangle = \frac{\left\langle \hat{\xi}^2 \right\rangle + \langle \hat{\eta}^2 \rangle}{\omega^2 + \gamma_m^2} = \frac{k_g \bar{b}_m \left(2\bar{b}_m + 1\right) + \gamma_m \hat{m}}{\omega^2 + \gamma_m^2} \tag{S21}$$

$$\begin{aligned} \sigma^2_{m,ss} &= \frac{k_g \bar{b}_m \left(2\bar{b}_m + 1\right) + \gamma_m m}{2\gamma_m} \\ &= \frac{1}{2}\bar{m}\left(2\bar{b}_m + 1\right) + \frac{1}{2}\bar{m} = \bar{m}\left(\bar{b}_m + 1\right). \end{aligned} \tag{S22}$$

From the burst Langevin equation we derived as in Eq. (21) in the main text, the steady-state variance is obtained with the mRNA burst size $\bar{b}_m$ included.

For the case of both mRNA and protein bursts:

$$\frac{dp}{dt} = k_g \bar{b}_m \bar{b}_p + \xi - (\gamma_p p + \eta) \tag{S23}$$

$$\langle \xi\xi' \rangle = k_g \bar{b}_m \bar{b}_p \left(2\bar{b}_m \bar{b}_p + 2\bar{b}_p + 1\right) \tag{S24}$$

$$\langle \eta\eta' \rangle = \gamma_p p. \tag{S25}$$

The steady-state variance is:

$$\delta\hat{p} = \frac{\hat{\xi} + \hat{\eta}}{i\omega + \gamma_p} \tag{S26}$$

$$\left\langle \delta\hat{p}^2 \right\rangle = \frac{\left\langle \hat{\xi}^2 \right\rangle + \langle \hat{\eta}^2 \rangle}{\omega^2 + \gamma_p^2} = \frac{k_g \bar{b}_m \bar{b}_p \left(2\bar{b}_m \bar{b}_p + 2\bar{b}_p + 1\right) + \gamma_p \hat{p}}{\omega^2 + \gamma_p^2} \tag{S27}$$

$$\begin{aligned} \sigma^2_{p,ss} &= \frac{k_g \bar{b}_m \bar{b}_p \left(2\bar{b}_m \bar{b}_p + 2\bar{b}_p + 1\right) + \gamma_p p}{2\gamma_p} \\ &= \frac{1}{2}\bar{p}\left(2\bar{b}_m \bar{b}_p + 2\bar{b}_p + 1\right) + \frac{1}{2}\bar{p} = \bar{p}\left(\bar{b}_m \bar{b}_p + \bar{b}_p + 1\right) \end{aligned} \tag{S28}$$

III. THE MODIFIED τ-LEAPING SCHEME FOR BURST PRODUCTION

Detailed considerations for τ selection are given in this section. A relative tolerance ϵ is first specified (as 0.03 in the present work) for the change of reaction propensities as suggested.[11] After the time step τ, the change in each reaction's propensity is required to be less than ϵ. In this way, an upper bound for τ can be obtained.

To select a τ, we follow the relations (Eqns. (32) and (33) in the work of Cao *et al*[11]):

$$\tau' = \min_{i \in I_{ncr}} \left\{ \frac{\max\{\epsilon x_i/g_i, 1\}}{|\mu_i|}, \frac{\max\{\epsilon x_i/g_i, 1\}^2}{\sigma_i^2} \right\} \tag{S29}$$

$$\mu_i = \sum_j \nu_{ij} a_j \tag{S30}$$

$$\sigma_i^2 = \sum_j \nu_{ij}^2 a_j. \tag{S31}$$

τ' is determined by either the first term with μ_i, the average change of x_i, or the second term with σ_i^2, the fluctuation of x_i. The state change number, ν_{ij}, is the change in x_i through the jth reaction with propensity a_j. $g_i \geq 1$ is a factor related to x_i's highest reaction order. If the reaction order is ≥ 2, x_i can be consumed quickly, thus, τ needs to be selected smaller with $g_i \geq 2$. For simplicity, we take the protein's $g_p = 1$ by assuming that for all reactions involving p, the highest reaction order is one, such as first-order degradation. When reactant number is small ($x_i \leq 33$ with $\epsilon = 0.03$), the numerator ($\max\{\epsilon x_i/g_i, 1\}$) makes sure that at least one-particle change is allowed. Only non-critical reactants (ncr) are considered in Eq. (S29), which means that the amount of x_is are large and remain similar after all possible reactions in a time step.

Critical reactants (cr) are those with number of molecules may become zero within ten reactions. Reaction propensities (a_j) involving critical reactants are summed up as

$$a_{0,cr} = \sum_{j \in J_{cr}} a_j. \tag{S32}$$

Another τ'' is estimated as in the Gillespie algorithm:

$$\tau'' = \frac{1}{a_{0,cr}} \ln\left(\frac{1}{1 - r_1}\right), \tag{S33}$$

where r_1 is a random number from an uniform distribution in the unit interval. τ is then selected:

$$\tau = \min\{\tau', \tau''\}. \tag{S34}$$

If $\tau = \tau''$, there will be one critical reaction, along with other non-critical reactions. On the other hand, when $\tau = \tau'$, there will be only non-critical reactions taking place. Following the scheme of Cao *et al*,[11] different reactions are classified into two groups, critical or not, and the smaller time step of the two is selected.

The above scheme[11] is based on the assumption that when reactions are non-critical, different reactions are independent and smallest time leap do not change the system much. When reactions are critical, the other method is used in selecting τ. However, the production is always non-critical whether in bursts and non-bursts, because production always increases particle number and the consumption of amino acids is not considered in the model. Furthermore, the burst production is a special case, because the protein number can be increased dramatically in a time step as short as a mRNA's lifetime. Followed by the burst production of certain genes, other reaction propensities are highly affected. Therefore, there is a need to reconsider the selection of τ in propagating the burst Langevin equation.

We consider the burst production as another reaction group, in addition to the original critical or non-critical groups. Following Eq. (S29), we reconsider the selection for τ' only for the burst production. As in the main text, we define the burst production's propensity $a_b = k_g \bar{b}_m \bar{b}_p$ and state change number $\nu_b = 1$. With expressions of μ_b and σ_b, τ' is determined as:

$$\tau' = \min\left\{\frac{\max\{\epsilon p, 1\}}{k_g \bar{b}_m \bar{b}_p}, \frac{\max\{\epsilon p, 1\}^2}{k_g \bar{b}_m \bar{b}_p}, \tau'_{j\in nb}\right\}, \tag{S35}$$

$$\mu_b = \sigma_b^2 = k_g \bar{b}_m \bar{b}_p \tag{S36}$$

where j is for other reactions of p, and $\tau'_{j\in nb}$ is determined from those reactions using Eq. (S29). We can see that τ' is highly dependent on $p(t)$ in the numerator. When protein number is large enough ($p > 33, \epsilon p > 1$), $\frac{\max\{\epsilon p,1\}}{k_g \bar{b}_m \bar{b}_p} \leq \frac{\max\{\epsilon p,1\}^2}{k_g \bar{b}_m \bar{b}_p}$, and thus, Eq. (S35) can be simplified as:

$$\tau' = \min\left\{\frac{\max\{\epsilon p, 1\}}{k_g \bar{b}_m \bar{b}_p}, \tau'_{j\in nb}\right\}. \tag{S37}$$

When τ' is selected as $\epsilon p / k_g \bar{b}_m \bar{b}_p$, the protein produced by burst production is equal to $\epsilon p = k_g \bar{b}_m \bar{b}_p \tau'$. When the burst size ($\bar{b}_p$) is small, several bursts can be included in τ' and when $\bar{b}_p$ is large, only a portion of a burst is introduced into the system. Therefore, when $p(t)$ is large enough, original scheme defined in Eq. (S29) works well to select a τ', either includes many burst events or just a portion of a burst.

When protein number is low ($p \leq 33$), τ is selected as $1/k_g \bar{b}_m \bar{b}_p$. Only one protein is allowed to produced in such τ. If $\bar{b}_p \geq 10$, it is very possible that the small τ will be selected $\bar{b}_p$ times consecutively for a burst event. And no other reactions happen in those tiny time

steps, and thus, the simulation time is wasted. To avoid this situation, we remove $\bar{b}_p$ from the denominator and select $\tau = 1/k_g\bar{b}_m$. In other words, we make τ being long enough for a complete burst event. Protein number produced in such τ is:

$$a_b\tau = k_g\bar{b}_m\bar{b}_p \times \frac{1}{k_g\bar{b}_m} = \bar{b}_p, \tag{S38}$$

which is a consistent result as the average protein number from a burst event. Therefore, when protein number is low, the selection of τ' can be modified accordingly as below:

$$\tau' = \min\left\{\max\left\{\frac{\epsilon p}{k_g\bar{b}_m\bar{b}_p}, \frac{1}{k_g\bar{b}_m}\right\}, \tau'_{j\in nb}\right\}, \tag{S39}$$

to include burst production.

There may be a definition conflict for the reaction propensity of burst. Previously, we define the burst's propensity $a_b = k_g\bar{b}_m\bar{b}_p$ and the state change number $\nu_b = 1$. Some may take burst propensity defined as $a_b = k_g\bar{b}_m$ and state change number $\nu_b = \bar{b}_p$, then σ_b^2 become $k_g\bar{b}_m\bar{b}_p^2$. If we only consider the case of $\bar{b}_p \geq 1$, very small τ will be selected as $\frac{\max\{\epsilon p,1\}^2}{k_g\bar{b}_m\bar{b}_p^2}$ from Eq. (S35), due to $\bar{b}_p^2$ in σ^2. When $\epsilon p \leq 1$, modifying the term $\frac{1}{k_g\bar{b}_m\bar{b}_p^2}$ as $1/k_g\bar{b}_m$ also helps to select a proper τ for the burst production.

When there are other reactions with higher order, original terms in Eq. (S29) are included, and the selection of τ' will be

$$\tau' = \min\left\{\max\left\{\frac{\epsilon p/g_p}{k_g\bar{b}_m\bar{b}_p}, \frac{1}{k_g\bar{b}_m}\right\}, \frac{\max\{\epsilon p/g_p, 1\}}{|\mu_{j\in nb}|}, \frac{\max\{\epsilon p/g_p, 1\}^2}{\sigma^2_{j\in nb}}\right\}. \tag{S40}$$

Furthermore, if there are many reactants x_i are included,

$$\tau' = \min_i\left\{\max\left\{\frac{\epsilon x_i/g_i}{k_{gi}\bar{b}_{mi}\bar{b}_{pi}}, \frac{1}{k_{gi}\bar{b}_{mi}}\right\}, \frac{\max\{\epsilon x_i/g_i, 1\}}{|\mu_{j\in nb}|}, \frac{\max\{\epsilon x_i/g_i, 1\}^2}{\sigma^2_{j\in nb}}\right\}. \tag{S41}$$

If x_i are not produced in bursts, the first term can be omitted in the above equation.

IV. SUPPLEMENTARY RESULTS

A. standard deviation comparison among different methods

In this work, protein's steady-state variance can be obtained by four methods. LNA is the first one that provides an exact analytical expression as in Eq. (S13). Gillespie algorithm is the second method, which is an exact numerical simulation method. We derived the burst

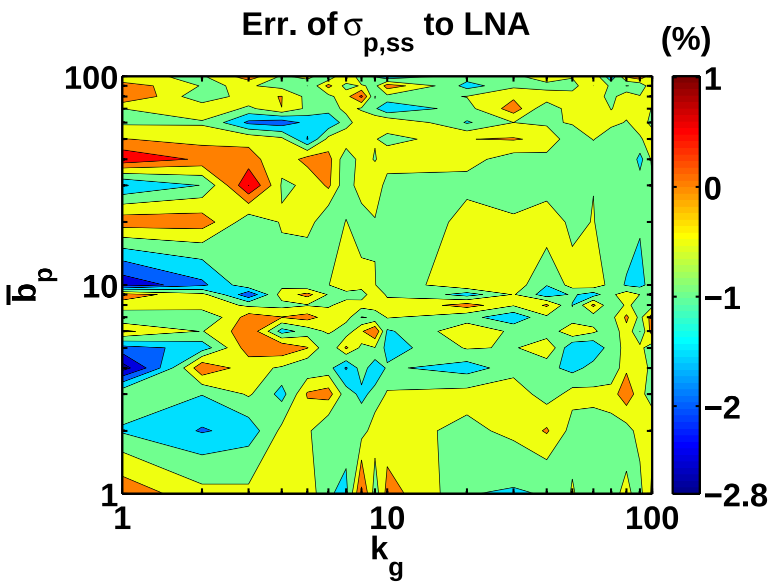

FIG. S1. The comparison between the Gillespie simulations and LNA. Shown are the normalized error of $\sigma_{p,ss}$ in % from the Gillespie simulations comparing to the square root of Eq. (S13) from the LNA with various gene activation rate k_g and average protein burst size $\bar{b}_p$. Statistics are in the steady state of 30,000 independent points for the model as defined in Eqs. (1) to (3) of the main text with parameters $k_m = 100$, $\gamma_g = 100$, $\gamma_m = 10$ and $\gamma_p = 1$.

Langevin equation and deduced an analytical expression for variance as in Eq. (S28), which is the third method. Finally, we also can obtain protein's steady-state variance from the numerical simulation following the burst Langevin equation. We want to show the differences among these four methods, especially the two methods based on the burst Langevin equation.

We compared the $\sigma_{p,ss}$ from the LNA and Gillespie algorithm first, which are both methods without any approximation. We collected 30,000 independent points of protein numbers from Gillespie simulation. In the parameter range that we tested, $\sigma_{p,ss}$ values as the squared root of Eq. (S13) from the LNA are from 2.84 to 757.07. Shown in Fig. S1 are the normalized error of $\sigma_{p,ss}$ from the Gillespie algorithm comparing to the squared root of Eq. (S13). We can see that $\sigma_{p,ss}$ from the Gillespie simulations and LNA are very close to each other. The normalized errors are $\leq 3\%$. Such errors are due to the randomness and finite samplings in simulations. Overall, LNA's variance expression and the Gillespie simulation can be both taken as the exact results.

Next, we compare the $\sigma_{p,ss}$ values as the square root of Eq. (S28) from the burst Langevin

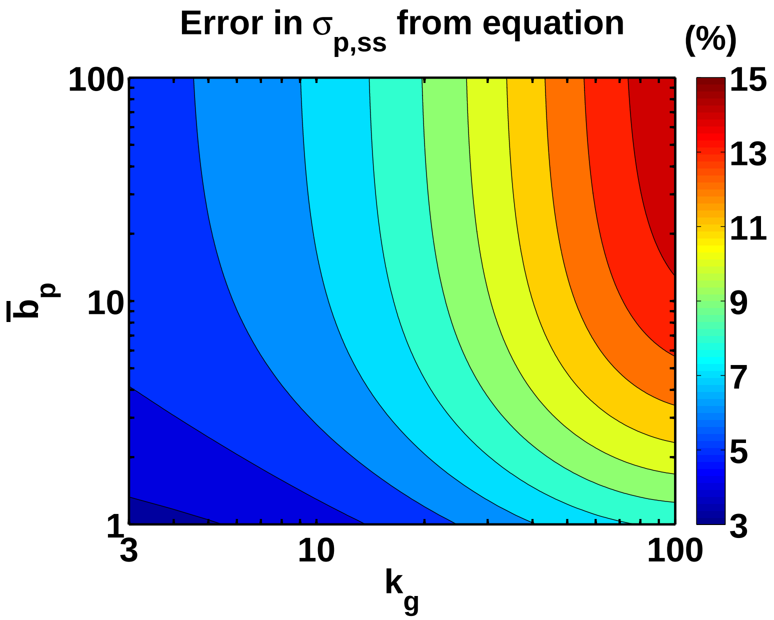

FIG. S2. Difference of $\sigma_{p,ss}$ (in %) between the analytical expression from the burst Langevin equation in Eq. (S28) and that in Eq. (S13) from the LNA with various gene activation rate k_g and burst size $\bar{b}_p$

equation to that from the exact expression in Eq. (S13) of LNA, and show in the Fig. S2. Due to the burst production approximation in Eq. (S28), it overestimates $\sigma_{p,ss}$ by 3% – 15%.

We check the accuracy in the standard deviation of protein's steady-state distribution from the numerical simulations following the burst Langevin equation by comparing to the Gillespie algorithm. In Fig. S3, we can see that the $\sigma_{p,ss}$ differences between the two simulations is from -13% to 14% mainly depending on k_g. When $k_g < 10$, the burst Langevin simulations underestimate $\sigma_{p,ss}$, while it overestimates in the region of large k_g.

From Fig. S2, we can see that $\sigma_{p,ss}$ from the analytical expression in Eq. (S28) (as $\sigma_{p,ana}$) is always an overestimation. From Fig. S3, $\sigma_{p,ss}$ from the numerical simulation following Eq. (31) (as $\sigma_{p,num}$) is not always an overestimation. For $\sigma_{p,ss}$, there are some differences between the analytical expression in Eq. (S28) and the numerical simulation, though both of them are based on the same burst Langevin equation in Eq. (31). In Fig. S4, we show the differences in $\sigma_{p,ss}$ between the two methods as:

$$\text{Difference in } \% = \frac{\sigma_{p,ana} - \sigma_{p,num}}{\sigma_{p,num}} \times 100\%. \tag{S42}$$

We can see that only when $k_g > 10$, the difference between two method become $< 5\%$.

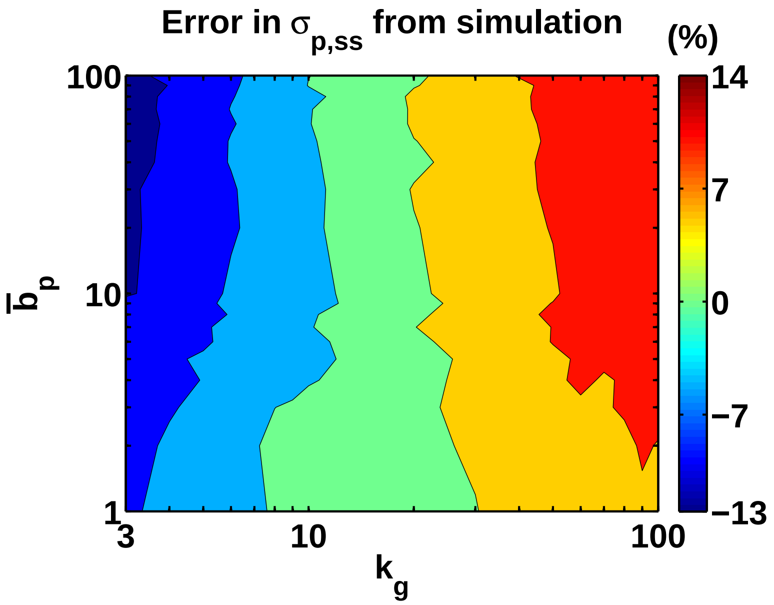

FIG. S3. Difference of $\sigma_{p,ss}$ between two numerical simulations. With various gene activation rate k_g and burst size $\bar{b}_p$, shown are the differences of $\sigma_{p,ss}$ in % from the burst Langevin simulation and that from the Gillespie algorithm. Statistics were taken with the same set up as in Fig. S1 for both simulations.

The "negative production" as seen in Fig. 8a of the main text may reduce $\sigma_{p,ss}$ from the numerical simulations. In the algorithm we proposed, when a negative production number is randomly selected, the simulation continues with no production until the accumulation of the serial production number becomes a positive number. Although such treatment keeps the same average production number, it decreases the variance of burst production, and consequently, the variance of the steady-state distribution is decreased. In Fig. S5, we showed the percentage of negative production with various k_g and $\bar{b}_p$. It is seen that there is always some possibilities to obtain negative production numbers. When $k_g < 10$, the large the negative production percentage reduces the overestimation of the analytical expression in Eq. (S28). When $k_g \geq 20$, the negative productions become $< 30\%$ as shown in Fig. S5, the variance from the numerical simulation and that from analytical expression become close to each other, as seen in the right-handed part of Fig. S4. Therefore, large negative production in the numerical simulation reduces the overestimation of the analytical expression in $\sigma_{p,ss}$.

To obtain an accurate $\sigma_{p,ss}$ from the burst Langevin simulation, we can select $k_g = 10-20$ where the difference of $\sigma_{p,ss}$ are close to zero, as shown in Fig. S3. Another approach is to

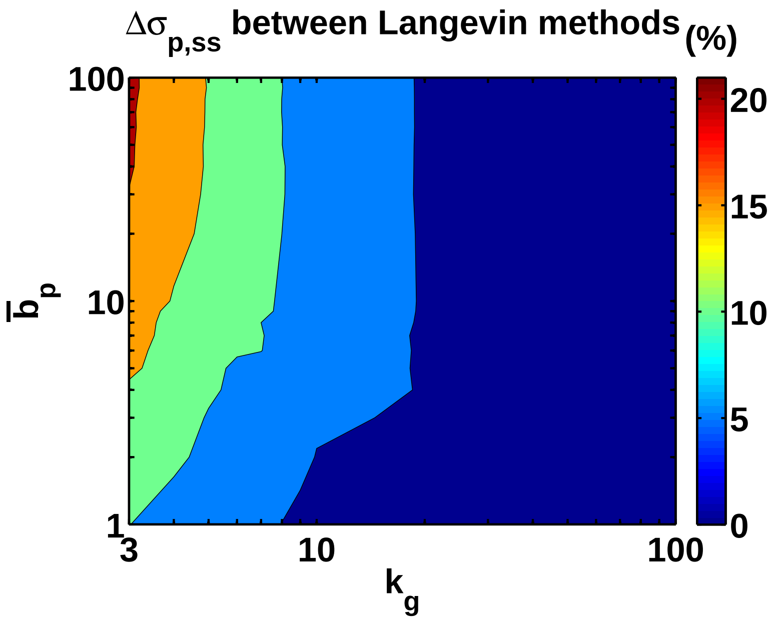

FIG. S4. Difference of $\sigma_{p,ss}$ between the analytical expression and the numerical simulation from the burst Langevin equation. With various gene activation rate k_g and burst size $\bar{b}_p$, shown are the differences of $\sigma_{p,ss}$ in % from the analytical expression in Eq. (S28) and that from the burst Langevin simulation.

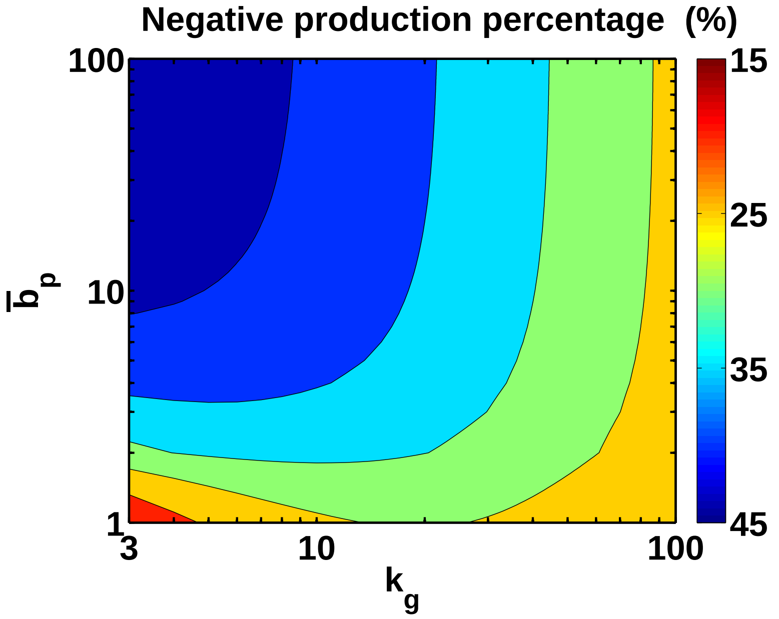

FIG. S5. Shown are the percentage of negative production distribution with various k_g and burst size $\bar{b}_p$ with fixed $\tau = 0.03\,(1/\gamma_p)$ and other parameter as defined in the caption of Fig. S1.

introduce an desirable scaling factor to rescale the noise strength in the burst production. Consequently, $\sigma_{p,ss}$ will be changed by the scaling factor. The differences in Fig. S3 suggest that the scaling factor highly depends on k_g. The scaling factor is also affected by $\bar{b}_p$ in a subtle way. However, with a pre-determined scaling factor in the production variance, the $\sigma_{p,ss}$ change from the numerical simulations may not be easily and directly estimated. Therefore, in order to have an accurate $\sigma_{p,ss}$, iterative tuning the scaling factor may be necessary.

B. k_g and $\bar{b}_m$ are more critical than $\bar{b}_p$ for the accuracy of $\sigma_{p,ss}$

We also included another test with various $\bar{b}_m$ and $\bar{b}_p$ about the accuracy of $\sigma_{p,ss}$ from the burst Langevin simulation. The results are shown in Fig. S6. We can see that with an increase in $\bar{b}_m$, the error of $\sigma_{p,ss}$ is also increases from -8.5% to 2.5%. However, such increase in error is not as much as compared to the increase shown in Fig. S3, which is from -13% to 14%, with an increase in k_g. On the other hand, both from Figs. S3 and S6, we can see the increase in $\bar{b}_p$ from 1 to 100 do not affect the accuracy of $\sigma_{p,ss}$. Varying k_g has the effect of multiplication on the noise of production, because k_g not only determines mRNA's burst frequency, but also determines the mRNA's burst size as in Eq. (16) in the main text. Burst size of mRNA, $\bar{b}_m$, is multiplied with $\bar{b}_p$ only, thus, it has less effect on the accuracy of $\sigma_{p,ss}$ than k_g. Finally, protein burst size, $\bar{b}_p$, is the least determinant kinetic parameter.

C. Significantly small k_g and γ_g leading to protein number's bimodal distributions

In Fig. S7, we included additional results from protein burst Langevin simulation comparing to the Gillespie algorithm in their distributions. It is seen that the burst Langevin simulation can produce similar bimodal distributions with different k_m and $\bar{b}_p$ pairs. In this case, the protein is produced in bursts, and our burst Langevin formulation is not affected by the slow gene state changes.

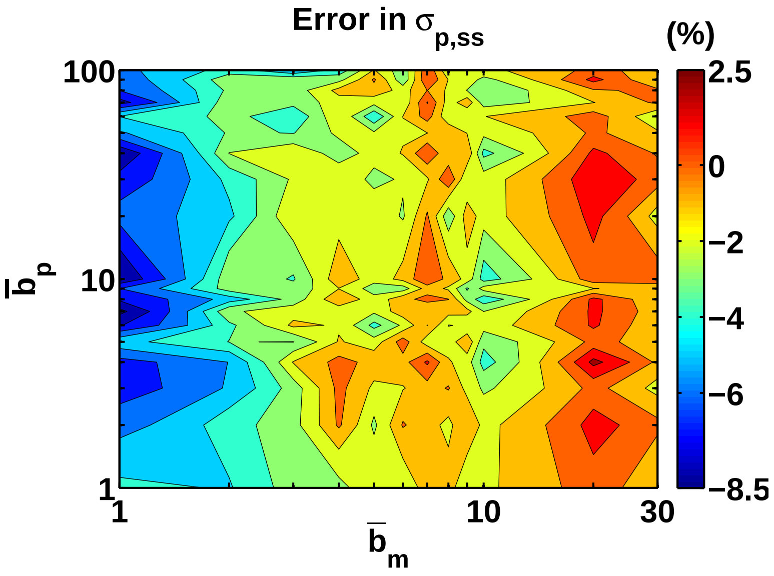

FIG. S6. The error of the burst Langevin simulation comparing to that from the Gillespie algorithm. Shown are errors in % of $\sigma_{p,ss}$ with various pairs of $\bar{b}_m$ and $\bar{b}_p$. k_m and k_p were determined by Eqs. (5) and (16) in the main text with given $\bar{b}_m$ and $\bar{b}_p$, with other parameters $k_g = 5$, $\gamma_g = 100$, $\gamma_m = 10$ and $\gamma_p = 1$.

D. Burst Langevin for non-linear activation

In Fig. S8, we included a case of non-linear activating regulation from the burst Langevin simulation comparing to the Gillespie algorithm to show that the burst Langevin simulation can produce accurate downstream $\bar{p}_2$ even with a non-linear regulation function. With all the parameter setting similar to that shown in Fig. 6 of the main text, we found that the error of $\bar{p}_2$ is from -3% to 5%, which is smaller than the case of repression (from -4% to 10%).

E. Genetic switching dynamics with different parameter sets

In Fig. S9, we included additional results for genetic switching dynamics to show that the burst Langevin simulation produces accurate dynamics with different parameter sets.

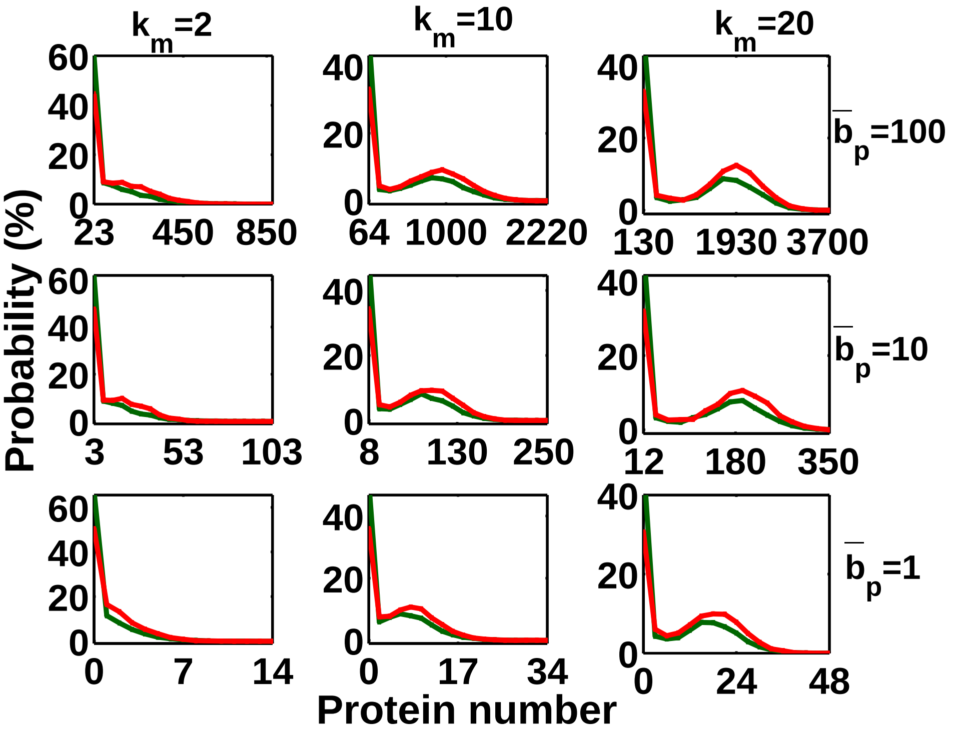

FIG. S7. Comparison of different algorithms producing protein's steady-state bimodal distributions. Shown are protein number distribution with different k_m and $\bar{b}_p$ from the endpoints of 10,000 independent stochastic trajectories using the Gillespie algorithm (green) and protein burst Langevin algorithm (red) with parameters fixed as $k_g = \gamma_g = 0.1$, $\gamma_m = 10$, and $\gamma_p = 1$.

REFERENCES

[1] N. Friedman, L. Cai, and X. S. Xie, Phys Rev Lett **97**, 168302 (2006).

[2] D. A. Mcquarri, J Appl Probab **4**, 413 (1967).

[3] D. T. Gillespie, Physica A **188**, 404 (1992).

[4] D. T. Gillespie, J Chem Phys **113**, 297 (2000).

[5] N. G. van Kampen, *Stochastic processes in physics and chemistry*, 3rd ed. (Elsevier, Amsterdam, 2007).

[6] J. Elf and M. Ehrenberg, Genome Res **13**, 2475 (2003).

[7] C.-C. S. Yan and C.-P. Hsu, The Journal of Chemical Physics **139**, 224109 (2013).

[8] R. Grima, Phys. Rev. E **92**, 042124 (2015).

[9] D. T. Gillespie, J. Comput. Phys. **22**, 403 (1976).

[10] D. T. Gillespie, J Phys Chem **81**, 2340 (1977).

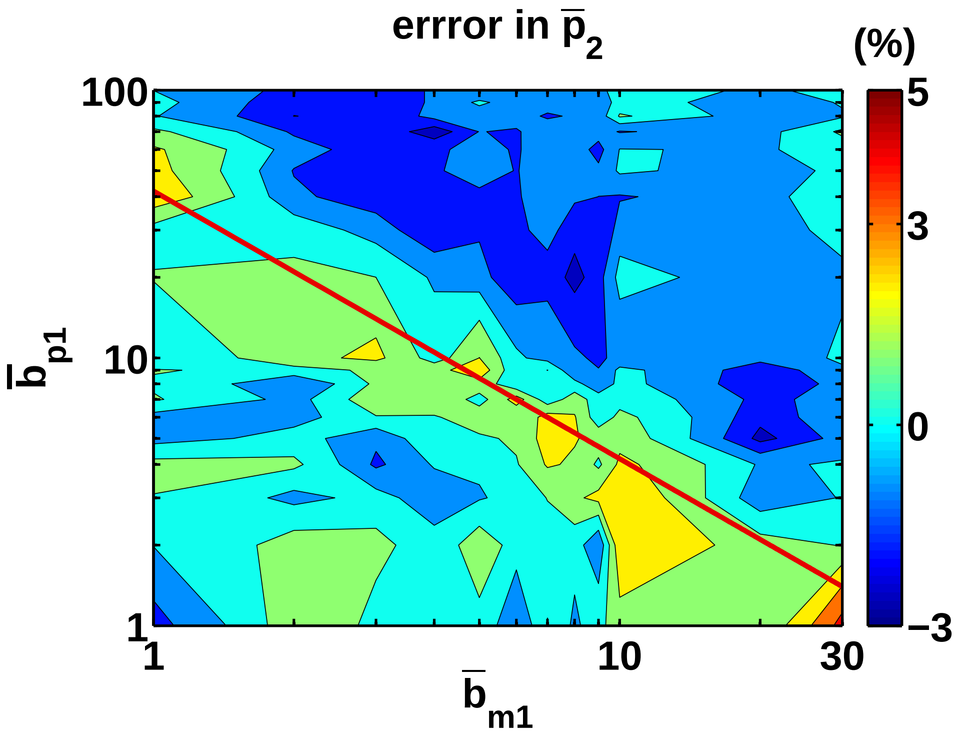

FIG. S8. A test of simulation error for gene expression under non-linear activation. Shown is the error in $\bar{p}_2$ from the burst Langevin simulation comparing to that from the Gillespie simulation. Here the p_1's burst frequency, $\bar{b}_{m1}$, and burst size, $\bar{b}_{p1}$, are varied over a range. Other parameters for p_1 are $k_{g1} = 5$, $\gamma_{g1} = 100$, $\gamma_{m1} = 10$ and $\gamma_{p1} = 1$. For p_2, the parameters are $k_{g2} = 5$, $\gamma_{g2} = 100$, $k_{m2} = 200$, $k_l = 60$, $\gamma_{m2} = 10$, $k_{p2} = 100$ and $\gamma_{p2} = 1$, $K = 200$ and $n_H = -3$ for activation. The red line in (b) corresponds to $\bar{p}_1 = K$.

[11] Y. Cao, D. T. Gillespie, and L. R. Petzold, J. Chem. Phys. **124**, 044109 (2006).
[12] J. M. Pedraza and A. van Oudenaarden, Science **307**, 1965 (2005).

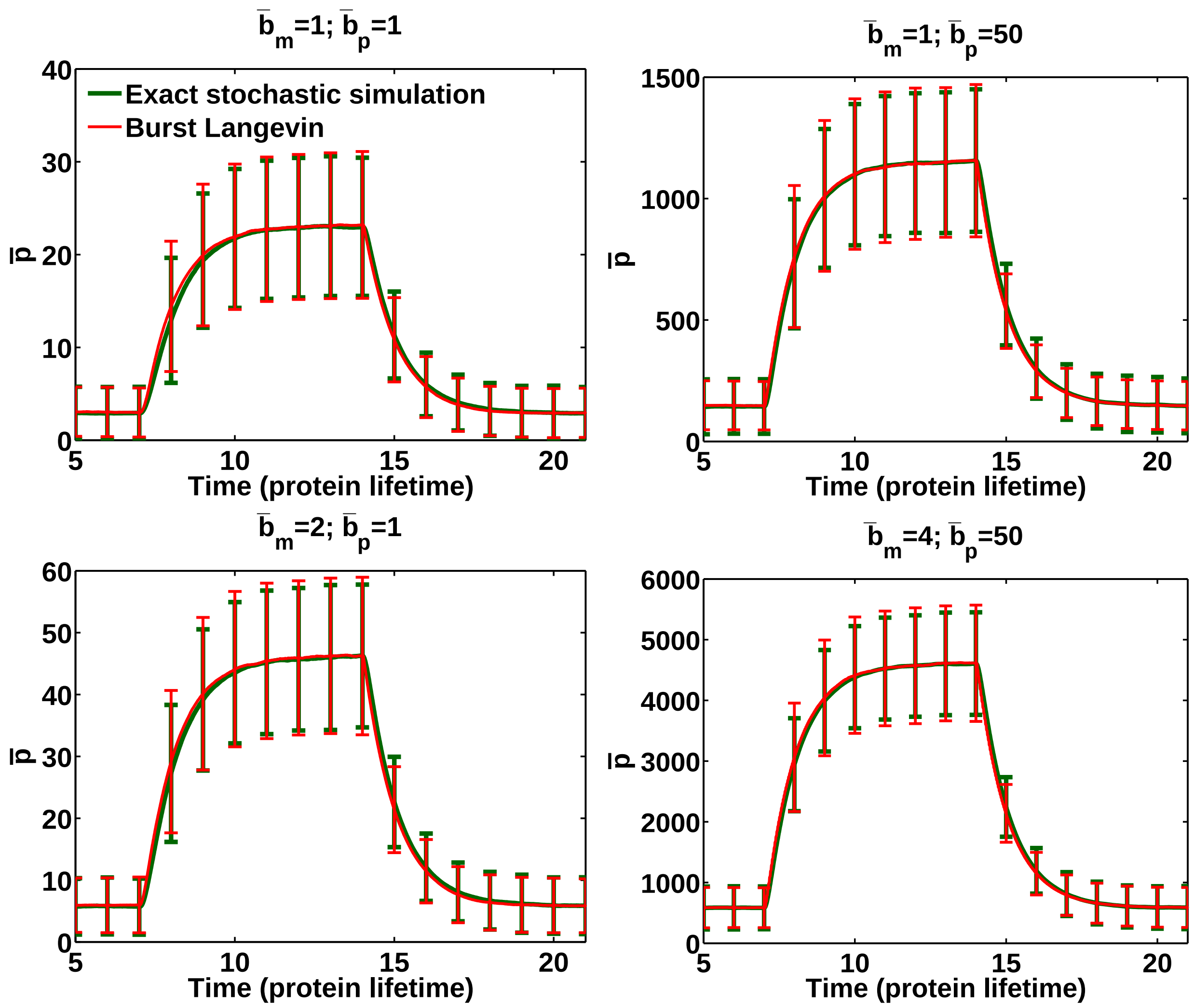

FIG. S9. Comparison of different algorithms for genetic switching dynamics. Shown are $\bar{p}$ with the distribution's standard deviation at different time from 10,000 independent stochastic trajectories using the Gillespie algorithm (green) and burst Langevin algorithm (red) with parameters $k_g = 30$ for $t = 7$ to 14; otherwise $k_g = 3$ and other parameters $\gamma_g = 100$, $\gamma_m = 10$, and $\gamma_p = 1$ with various pairs of $\bar{b}_m$ and $\bar{b}_p$ shown in each panel's title.

TABLE

TABLE S1: list of symbols

symbol	meaning	first appearance
$\bar{\square}$	average of burst event e, burst size b, or species $i = g, m, p, x, y$	Eq. (4)
$\hat{\square}$	variables in Fourier space	Eq. (S20)
$\langle\square\rangle$	take average	Eq. (10)
$\mathbf{A}$	Jacobian matrix	Eq. (S6)
$\mathbf{A}^T$	transpose of Jacobian matrix	Eq. (S6)
$\mathbf{D}$	diffusion matrix	Eq. (S6)
K	threshold in Hill function	Eq. (41)
$\mathcal{N}_j(0,1)$	unity Gaussian function for reaction j	Eq. (9)
$P(b_m)$	probability function for mRNA burst size	Eq. (15)
$P(i;t)$	probability function in CME	Eq. (S3)
$\mathbf{V}$	variance matrix	Eq. (S5)
a_0	total reaction propensity sum of all reactions	Eq. (S14)
a_j	reaction propensity for jth reaction	Eq. (43)
$\bar{b}_i$	averaged burst size for species $i = m, p, y$	Eq. (4)
b_m	possible burst size ≥ 0 for mRNA	Eq. (15)
b_{yl}	burst size for species y from lth event	Eq. (9)
dt	the time propagated in the Gillespie algorithm with one reaction	Eq. (S15)
$\bar{e}_i$	averaged burst event number in τ for $i = m, p, y$	Eq. (10)
e_i	burst event number in τ for $i = m, p, y$	Eq. (9)
g	gene's activity	Eq. (1)
g_i	scaling factor of ϵ for x_i	Eq. (S29)
i	index for species	Eq. (43)
j	index for reactions	Eq. (42)
k_i	production or activation rate constant for species $i = g, m, p, x, y$	Eq. (1)
k_l	leaking (basal) transcription rate constant for species p_2	Eq. (41)
l, l'	index for different burst event	Eq. (9)

Continued on next page

TABLE S1: list of symbols – continued from previous page

symbol	meaning	first appearance
m	mRNA copy number	Eq. (2)
n	copy number of gene	after Eq. (13)
n_H	Hill coefficient	Eq. (41)
p	protein copy number	Eq. (3)
q	probability for no mRNA production in a gene activation period	Eq. (15)
r_1, r_2	random number from a uniform distribution from zero to one	Eq. (S15)
x	short-lived species in the general burst model	Eq. (6)
x_i, x_k	all reactants in a system	Eq. (S9)
y	species with burst production in the general burst model	Eq. (7)
Δ_i	averaged production number in τ for $i = m, p, y$	Eq. (9)
δi	$\delta i = i(t) - \bar{i}$	Eq. (S5)
ϵ	tolerable reaction propensity change	Eq. (43)
η	degradation noise strength in a Langevin equation	Eq. (S17)
γ_i	degradation or deactivation rate constant for species $i = g, m, p, x, y$	Eq. (1)
ν_{ij}	the number x_i changed through one reaction j	Eq. (43)
σ_{bi}^2	variance of burst size distribution for $i = m, p, y$	Eq. (11)
σ_{ei}^2	variance of burst event distribution for $i = m, p, y$	Eq. (11)
σ_{Δ_i}	production distribution's standard deviation in τ for $i = m, p, y$	Eq. (9)
$\sigma_{i,ss}$	standard deviation of $i = m, p$ in a steady state	Eq. (23)
τ	a longer time step than dt allowing more reactions	Eq. (9)
ξ	production noise strength in a Langevin equation	Eq. (S17)
